# Species identification of Bombyx mori and Antheraea pernyi silk via immunology and proteomics

**DOI:** 10.1038/s41598-019-45698-8

**Published:** 2019-06-28

**Authors:** Jincui Gu, Qingqing Li, Boyi Chen, Chengfeng Xu, Hailing Zheng, Yang Zhou, Zhiqin Peng, Zhiwen Hu, Bing Wang

**Affiliations:** 1 Key Laboratory of Advanced Textile Materials and Manufacturing Technology, Ministry of Education, Zhejiang Sci-Tech University, Hangzhou, 310018 China; 2 Key Scientific Research Base of Textile Conservation, State Administration for Cultural Heritage, China National Silk Museum, Hangzhou, 310002 China; 3 Institute of Textile Conservation, Zhejiang Sci-Tech University, Hangzhou, 310018 China

**Keywords:** Biochemical assays, Assay systems

## Abstract

In recent years, increasing attention has been paid to the origin, transmission and communication of silk. However, this is still an unsolved mystery in archaeology. The identification of silk-producing species, especially silk produced by *Bombyx mori* (*B. mori*) and *Antheraea pernyi* (*A. pernyi*), is of key significance to address this challenge. In this study, two innovative methods, i.e. immunology and proteomics, were proposed and successfully established for the species identification of silks. ELISAs result demonstrated that the two prepared antibodies exhibited high sensitivity and specificity in distinguishing *B. mori* and *A. pernyi* silk. No cross-reactivity with each other was observed. Moreover, biomarkers were obtained for *Bombyx* and *Antheraea* through proteomic analysis. It was also confirmed that the biomarkers were suitable for identifying the species that produced a given silk sample. Compared with conventional methods for distinguishing silk species, immunological and proteomics techniques used in tandem can provide intact information and have the potential to provide accurate and reliable information for species identification.

## Introduction

Silk, a symbol of ancient Chinese culture, is the best-known textile material and was spread around the world along the Silk Road^1^. The ancestors of the Chinese spun raw silk as early as the Neolithic Age. Silk was derived from wild silkmoth species, rather than *Bombyx mori (B. mori)*, until the wild silkworms were domesticated. Among wild silkworms, the cocoon of *Antheraea pernyi (A. pernyi)* is the most widely used and described. It is generally believed that silk produced by *B. mori* was transported from China to Western Asia and the Mediterranean after the opening of Silk Road in the Western Han Dynasty (206 B.C.-8 A.D.). However, the first example of wild *Antheraea* silk in the Indus civilization has been dated to 2450–2000 BC^2^. These findings have encouraged people to reconsider the origin and transmission of ancient silks. Thus, the identification of ancient traces of silk produced by *A. pernyi* and *B. mori* has become of great significance in the archaeological field.

A single silk glued with sericins consists of 70–80% fibroins^3^, whose amino acid sequence reflects the diverse morphology and structure of silk^4,5^. Fibroin derived from different silk also imparts the specific desirable performance properties of silk, such as softness, lightness, and smoothness^6^. Thus, studying the fibroin differences between *B. mori* and *A. pernyi* silk is a basis for identifying their species. In the past several decades, various studies regarding silk characterization have been carried out using a variety of methods, such as scanning electron microscopy (SEM)^7^, Fourier transform infrared spectroscopy (FTIR)^8–10^ and amino acid analysis^11^. These methods can distinguish well-preserved silk from other fibers and identify what species produced the silk. However, silk is easily affected by light, radiation and temperature, which is particularly problematic for very old silk samples^12^. Thus, silk unearthed in graves usually suffers from physical and chemical changes, showing serious degradation and aging. Consequently, despite lots of meaningful information can be achieved by conventional methods, the efficacy is likely to be limited in the detection of ancient samples with contamination or severe degradation. Therefore, novel approaches for identifying the species that produced a given sample of silk should be developed.

Among the new rising technologies, immunological techniques and proteomics are preferred methods. More accurate information about ancient silks at the sequence level can be obtained using immunological techniques and proteomics. Both the two methods are particularly suitable for the identification of poorly preserved ancient silks (severely degraded or contaminated) or even the silk traces, as long as partial amino acid sequences are still present. This is also the key challenge facing researchers in archaeological sites.

Immunological techniques, including various efficient methods such as immunoprecipitation (IP), enzyme-linked immunosorbent assays (ELISAs), immuno-fluorescence microscopy and immunochromatography, present the advantages of high sensitivity, short processing times and low cost^13^. Therefore, immunological techniques have attracted increasing attention from professionals around the world. ELISA is the most commonly used of these methods and is widely applied in many fields^14–19^. In ELISA test, the antigen interacts with the primary antibody, and the antigen-antibody conjugates then combine with the secondary antibody. Thereafter, the reaction can be detected using a microplate reader. The preparation of the primary antibody is the most important step for immunoassays. The more specific the primary antibody, the more reliable the ELISA result. To design specific antibodies, the amino acid sequences of *B. mori* silk and *A. pernyi* silk were compared and the diagnostic sequences were selected for peptide synthesis. Immunospecific primary antibodies were produced by injecting rabbits with the synthesized peptide coupled with a carrier protein. Thus, it was feasible to use ELISA to identify the silk produced by *B. mori* or *A. pernyi*.

Proteomics is a sensitive, precise and high-throughput tool for understanding complex mixtures at the protein level^20,21^. This approach has recently been broadly adopted in many fields^22,23^. In particular, researches regarding proteomics applying in archaeology have been reported sporadically. For example, Mai *et al*. analyzed red cosmetic sticks in early Bronze Age at Xiaohe Cemetery using proteomics^24^. Shevchenko *et al*. identified the composition and manufacturing recipe of the 2500-year old sourdough bread from Subeixi cemetery in China via proteomics^25^. Solazzo *et al*. also used proteomic technology to identify protein remains in archaeological potsherds^26^. The results of these studies indicated that proteomic analysis is suitable not only for biology but also for archaeology. Proteomic technique mainly consists of two analysis strategies: two-dimensional gel electrophoresis and mass spectrometry. In-gel digestion is a classic approach for MS analysis, which has frequently been used to detect proteins in these years^27–29^. In addition, a correlative method based on gel-free digestion of protein followed by liquid chromatography coupled to tandem mass spectrometry (LC-MS/MS) analysis can now be used to achieve rapid development^30,31^.

**Figure 1:**
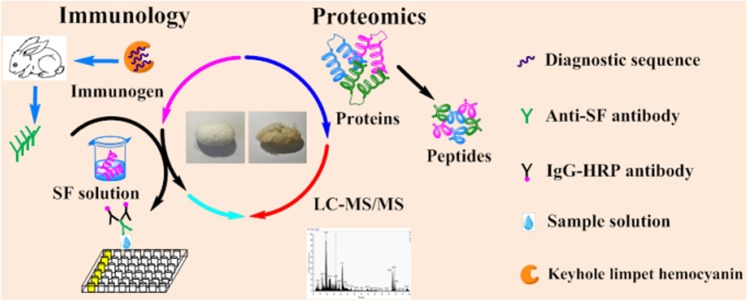
Experimental design for identifying species of silks produced by *B. mori* and *A. pernyi* via immunology and proteomics.

In the present study, we differentiate silk produced by *B. mori* and *A. pernyi* based on morphology and primary structure by combining traditional methods with immunological techniques and proteomics (Fig. 1). First, the silk was characterized via SEM and FTIR. Then, the amino acid composition of the silk was determined through amino acid analysis. Thereafter, an ELISA system was established to detect the corresponding fibroin in silk using diagnostic antibodies. To further distinguish *B. mori* silk from *A. pernyi* silk at the sequence level, an LC-MS/MS approach was utilized to elucidate the differences in their amino acid sequences and identify biomarkers for the silks. As we expected, the results revealed that the ELISA and proteomics approaches that we employed can provide accurate and reliable information. These findings probably have great potential for future applications.

## Materials and Methods

### Reagents and materials

HPR conjugated goat anti-Rabbit IgG (H + L) (100 *µ*g at 2 mg/mL) and the TMB color system were purchased from Hangzhou Hua’an Biotechnology Co., Ltd. Bovine serum albumin (BSA), sodium deoxycholate and ammonium bicarbonate were purchased from Sigma-Aldrich. Copper hydroxide was supplied by Shanghai Macklin Biochemical Co., Ltd. Ethanediamine, diethyl ether, methanol, acetone and hydrochloric acid were supplied by Tianjin Gaojing Fine Chemical Co., Ltd. Dithiothreitol (DTT), iodoacetamide (IAA), trifluoroacetic acid (TFA), formic acid (FA), sodium deoxycholate (SDC) and acetonitrile (ACN) were purchased from Thermo Fisher Scientific. The water used in all experiments was purified using a TPM Ulrapure water system. The silk cocoons (*B. mori* and *A. pernyi*) and silk fabrics (Samples I and Sample II) were kindly provided by the China National Silk Museum.

### Morphology

Images of *B. mori* silk cocoon, *A. pernyi* silk cocoon, and silk samples were obtained using a digital camera (Canon EOS700D). The cross-sectional morphology of the silk fibers was characterized with a polarizing microscope (AxioCam MRc 5). Samples were cut using a fiber slicer. The longitudinal surfaces of the samples were characterized via SEM (JEOL JSM-5610). The samples were adhered to conductive adhesives, sputter-coated with gold for 60 s and then examined via SEM at an extra-high tension of 1 kv.

### Chemical structure

Attenuated total reflection-Fourier transform infrared (ATR-FTIR) spectroscopy analysis (Thermo Scientific) was employed to characterize the main chemical bond of the silk in the wavenumber range of 500–4000 cm^−1^. Origin 9.0 software was applied to process the data.

### Amino acid analysis

The amino acid composition of the samples was characterized using an amino acid analyzer (AAA, Waters 2695). Samples were hydrolyzed with 6 M hydrochloric acid for 24 h at 110 °C to yield individual α-amino acids. After drying the hydrated solution under nitrogen, the hydrolyzate and the internal standard were dissolved in derivation liquid. The mass fraction and the molar fraction of the amino acids were calculated by AAA.

### Extraction of silk fibroin

Considering that *A. pernyi* silk is very difficult to dissolve in traditional extraction solutions (calcium chloride aqueous solution or calcium chloride-water-ethanol solution), a new approach was adopted for extracting silk fibroin. The detailed procedures performed were as follows.

After peeling the husks from the cocoons, the cocoons were incubated in a 0.5% (w/w) Na_2_CO_3_ aqueous solution at 98 °C for 1 h at a bath ratio of 1:100. After repeating this process 4 times, the degummed cocoon layer was soaked in 1% (w/w) hydrochloric acid at 25 °C for 1 h, then washed with water and dried at 80 °C. Next, the desiccated silkworm layer was soaked in 70% (w/w) methanol at 60 °C for 2 h and then placed in absolute ethanol at 60 °C for 1 h. The sample was subsequently soaked in acetone at 40 °C for 1 h, followed by immersion in ether at 25 °C for 1 h. After drying, the silk was soaked in a 100-fold volume of distilled water at 98 °C for 1 h, which was repeated 4 times until no biuret reaction occurred^32^.

Next, a solution was produced by dissolving 6.6 g of copper hydroxide in 50 mL of distilled water, to which 8.5 mL of ethylenediamine was added, and the mixture was stirred well to dissolve all solids before being brought to 100 mL distilled water. The silk sample was subsequently placed in the prepared copper ethylenediamine solution (1:50) and held at 60 °C for 1 h. After cooling to 25 °C, the pH of the solution was adjusted to 8.5 with 10% acetic acid on a magnetic stirrer. Then, the solution was purified using a dialysis membrane with a molecular weight cut off of 8 kDa. After dialyzing for 3 days, the resultant solutions were lyophilized to obtain silk fibroin powders of *B. mori* silk and *A. pernyi* silk, respectively (abbreviated as SF-BM and SF-AP).

0.1 g of silk fabrics were added to the extracting solution (copper ethylenediamine solution) at the bath of 1:50 and held at 60 °C for 1 h. The pH of the mixture was adjusted to 8.5 with 10% acetic acid on a magnetic stirrer and then centrifuged for 10 min at 4500 rpm. The supernatant was obtained for further identification.

### Preparation of primary antibodies

The immunogens used to generate specific primary antibodies were well-designed and carefully prepared. First, the diagnostic sequence (hapten) was selected by comparing the preliminary sequence differences of silk fibroin derived from *B. mori* silk and *A. pernyi* silk. (Fragmented amino acid sequences of silks produced by different species were registered in NCBI public databases (http://www.ncbi.nlm.nih.gov/pubmed/)). The MQRKNKNHGILGKC sequence was selected for *B. mori* silk^33^, while the CSHSHSYEASRISVH sequence was selected for *A. pernyi* silk. The selected peptides were synthesized using a peptide synthesizer (CS Bio). After coupling the synthetic peptide with keyhole limpet hemocyanin (Sangon, Shanghai), the immunogen (complete antigen) was used to induce specific primary antibodies via animal immunization. The process of animal immunization is described in detail in a previous report by our group^34^. The resulting anti-SF-BM and anti-SF-AP antibodies were further purified using a Protein A column and stored at −20 °C for use (anti-SF-BM antibody: 0.58 mg/mL; anti-SF-AP antibody: 0.33 mg/mL).

### Indirect ELISA procedures

Samples were dissolved in carbonate buffer (CB) at pH 9.6, and the concentration of the silk fibroin solution was then adjusted to 10 *µ*g/mL. In this indirect ELISA test, 96-well microplates were used and each column of the microplate was removable. In the microplates, the antigen was combined with the primary antibody, which was targeted by an enzyme-conjugated secondary antibody instead of being conjugated with the enzyme directly. The following section presents a detailed description of this procedure.

First, 100 *µ*L of the pretreated silk fibroin solution (10 *µ*g/ml) was added to each well, followed by incubation at 4 °C overnight until the antigen was attached to the plastic. Then, the solution was removed, and the plate was washed three times with 200 *µ*L of PBS. Next, 200 *µ*L of blocking buffer was added to each well, followed by incubation at 37 °C for 2 h to bind non-specific binding sites. The blocking buffer was removed, and the plate was washed again with 200 *µ*L of PBS three times. Then, 100 *µ*L of the primary antibody was added to each well, followed by incubation at 37 °C for 1 h. Thereafter, the solution was removed, and the plate was washed with 200 *µ*L of PBS three times. Next, 100 *µ*L of the secondary antibody (HPR-conjugated goat anti-rabbit IgG (H + L)) was added to the well, followed by incubation at 37 °C for 1 h. After removing the secondary antibody and washing five times with PBS, 100 *µ*L of substrate solution (TMB color system) was added to each well in a dark environment, where the plate was left at room temperature for 10 min. Finally, 100 *µ*L of 1 mol/L H_2_SO_4_ was added to end the color reaction, and sample absorbance was measured using a microplate reader (Bio-Rad model 550) at λ = 450 nm.

To ensure the accuracy of the experiment, a battery of controls were set up. Silk fibroin solution was employed as a positive control, and the antigen was replaced by CB (pH = 9.6) as a negative control to ensure that the antibody combined only with antigen. The cut-off value was defined as the mean OD_450_ of CB (pH = 9.6) plus three standard deviations (mean + 3 SD).

### Sample preparation for proteomics

To identify the silk fibroins of *B. mori* and *A. pernyi* at the primary structural level, a proteomic method was applied. Silk fibroins were first disintegrated in 100 *µ*L of lysate (4% SDC, 50 mM Tris-HCl pH = 8.0) in an Eppendorf tube. The fibroin concentration was measured using the Bradford method^35^. Then, 25 *µ*g of fibroins was added to 50 *µ*L of 50 mM NH_4_HCO_3_, followed by adding 5.5 *µ*L of 100 mM DTT and incubating at 37 °C for 1 h. Then, the sulfhydryl functional groups of silk fibroins were alkylated with 6 *µ*L of 500 mM IAA via incubation in the dark for 30 min. Thereafter, the samples were centrifuged in an ultrafiltration tube (10 kDa) for 15 min at 14,000 g and washed twice with 50 mM NH_4_HCO_3_ (containing 0.4% SDC). Next, after transferring to a new Eppendorf tube, the silk fibroins were digested in a mixture solution of 50 *µ*L of 50 mM NH_4_HCO_3_ (containing 0.4% SDC) and 2 *µ*L of 0.5 *µ*g/*µ*L trypsin for 14 h at 37 °C. The resulting tryptic peptides were recovered via ultrafiltration at 14,000 g for 15 min and then washed with 50 mM NH_4_HCO_3_. Next, 5% TFA was added to the samples, and they were centrifuged for 2 min at 14,000 g to remove SDC. Thereafter, the supernatant was desalted using Pierce^TM^ C18 spin columns. Finally, the samples were eluted twice with 50 *µ*L of 70% acetonitrile (ACN) and 0.1% FA in an ultrafiltration tube and lyophilized.

### Sample analysis and database searches

Tryptic peptides were separated in a Thermo Fisher Scientific EASY-nLC 1000 UPLC system using a C18 trap column (5 *µ*m, 100 Å, 2 cm × 100 *µ*m) and a C18 analytical column (2 *µ*m, 100 Å, 15 cm × 50 *µ*m). The peptides were eluted using a gradient of solvent A (0.1% FA in water solution) from 97% to 5% and a corresponding gradient of solvent B (0.1% FA in acetonitrile solution) from 3% to 95%, at a flow rate of 250 nL/min over 90 min. The UPLC system was directly interfaced with a Thermo Scientific Q-exactive mass spectrometer which equipped with a nanoelectrospray source. Mass spectrometry analysis was performed in the data-dependent acquisition mode. There was a single full-scan mass spectrum in the orbitrap (300–2000 m/z, 70000 resolution) followed by 20 data-dependent MS/MS scans at 27% normalized collision energy.

Raw MS files were analyzed using the Xcalibur 2.2 SPI system. The MS/MS spectra were searched against UniProt database (http://www.uniprot.org/uniprot/) using Proteome Discoverer software (Version PD1.4, Thermo Scientific, USA). Precursor ion masses between 350 Da and 8000 Da were searched, and the precursor mass tolerance was set to 10 ppm. The fragment mass tolerance was set to 0.02 Da. Peptide identification was filtered at 1% false discovery rate. The search included two modifications: oxidation and carbamidomethyl. The peptide length was set at 7–30 amino acids.

## Results and Discussion

### Morphology

**Figure 2:**
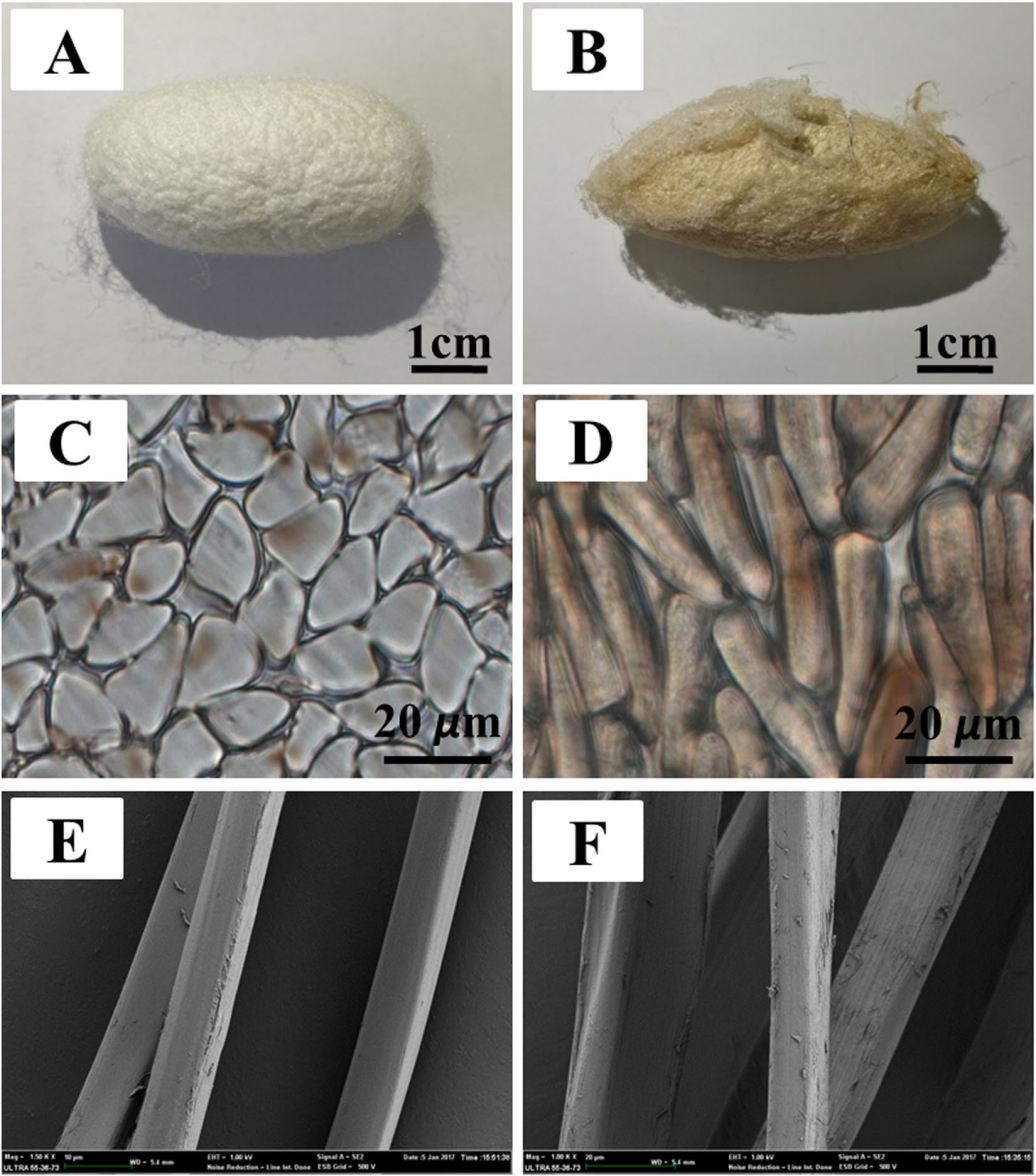
Morphology of silks produced by *B. mori* (left column) and *A. pernyi* (right column). (**A**,**B**) Digital images of cocoons; (**C**,**D**) Cross-sections of degummed silk fibers; (**E**,**F**) SEM images of the longitudinal surface.

Figure 2 shows images of *B. mori* (left column) and *A. pernyi* (right column) cocoons. The differences between the silks from the two species regarding their morphology, cross-section and longitudinal surface can be observed by comparing the images.

As shown in Fig. 2A,B, the cocoon of *B. mori* was oval, shiny and exhibited white color. The *A. pernyi* cocoon exhibited tawnier color and was more pointed at both ends. Compared with the *B. mori* cocoon, the cocoon of *A. pernyi* was more resistant to degumming. Cross-sections of *B. mori* silk and *A. pernyi* silk are displayed in Fig. 2C,D. The shape of *B. mori* silk was similar to an acute triangle, whereas *A. pernyi* silk was more rectangular, as verified by the SEM images shown in Fig. 2E,F. According to the magnified SEM images, the silk of *B. mori* was thinner and smoother than the silk of *A. pernyi*. In summary, *B. mori* silk and *A. pernyi* silk exhibit overt differences in morphology, which typically influences fiber mechanics.

### Chemical structure

**Figure 3:**
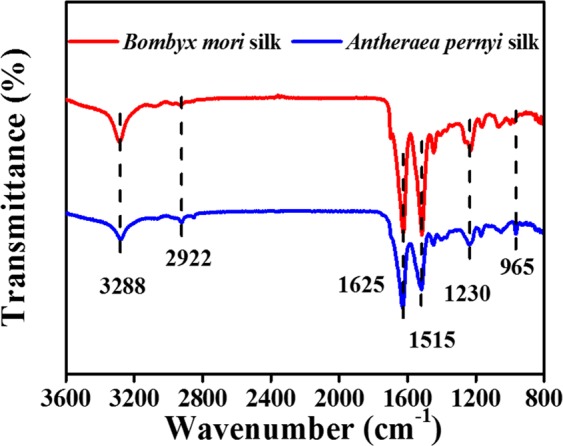
FTIR spectra of silks produced by *B. mori* and *A. pernyi*.

ATR-FTIR analysis was used to identify the functional groups of the degumming silks^36^. The characteristic peaks of the silks are shown in Fig. 3. Both samples displayed a peak at 3288 cm^−1^, with the characteristic signal from N–H stretching vibration coming from the amide A and amide B zones. The peak at 2922 cm^−1^ was assigned to the –CH_2_– vibration region. The spectra also showed peaks corresponding to amide bonds: the peak approximately 1583–1670 cm^−1^ corresponded to the amide I bond; that at 1474–1583 cm^−1^ to the amide II bond; and that at 1192–1295 cm^−1^ to the amide III bond. Differences between *B. mori* silk and *A. pernyi* silk could be observed in the spectra: there was a peak that was present only in *A. pernyi* silk at 965 cm^−1^. This peak was attributed to the peptide structure alanine-alanine (Ala-Ala), which rarely existed in *B. mori* silk^37^. Thus, the characteristic peak could be used to identify the silk species. However, the results will become unreliable for contaminative silk because miscellaneous peaks in the spectra might lead to analysis errors.

### Amino acid composition

The amino acid composition was analyzed, and the obtained mass fraction and molar fraction of the amino acids of the silk fibroins are shown in Table 1. The different silks exhibited different amino acid compositions. According to the results shown in Table 1, there were seventeen amino acids detected in the two silk fibroins. Consistent with the results of a previous study^38^, histidine (His) was present mainly in *A. pernyi* silk and absent from *B. mori*. Similarly, *B. mori* silk lacked cysteine (Cys), which was present in *A. pernyi* silk. The mass fraction of aspartic acid (Asp) in *A. pernyi* silk was 8.44%, whereas in *B. mori* silk, the percentage was only 2.86%. Alanine (Ala), glycine (Gly), serine (Ser) and tyrosine (Tyr) were the main amino acids found in both *B. mori* and *A. pernyi* silk, with total mass fraction of 83.35% and 74.59%, respectively. The crystalline degree of silk fibroin was determined by the contents of Gly, Ala, Ser and threonine (Thr)^39^. The higher content of these amino acids in *B. mori* silk suggests that this silk has higher degree of crystallinity than *A. pernyi* silk.

**Table 1:** Comparison of the amino acid composition of *B. mori* and *A. pernyi* silk.

Amino acid composition	*B. mori*silk		*A. pernyi* silk	
	wt^a^/%	X^b^/%	wt^a^/%	X^b^/%
Asp	2.86	2.05	8.44	6.35
Ser	13.36	12.12	12.34	11.76
Glu	2.47	1.6	2.17	1.48
Gly	34.24	43.48	20.69	27.61
His	0	0	2.46	1.59
Arg	1.29	0.7	5.65	3.25
Thr	1.35	1.08	1.63	1.37
Ala	25.23	27	31.57	35.87
Pro	0.81	0.67	0.75	0.65
Cys	0	0	0.17	0.14
Tyr	10.52	5.54	9.99	6.86
Val	3.63	2.95	1.1	0.94
Met	0.02	0.01	0.32	0.22
Lys	0.7	0.45	0.95	0.65
IIe	1.19	0.86	0.61	0.47
Leu	0.9	0.65	0.61	0.47
Phe	1.43	0.83	0.54	0.32

^a^wt: The mass fraction.

^b^X: The molar fraction.

### Species identification of silks via immunology

#### Optimum antibody dilution

**Figure 4:**
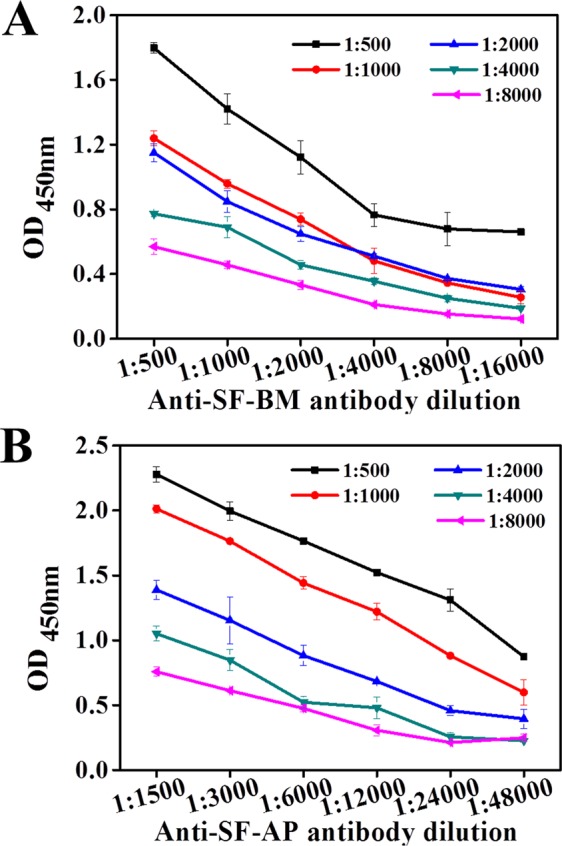
Indirect ELISA curves of secondary antibodies against primary antibodies at different dilutions. (**A**) ELISA curves of the secondary antibody against the anti-SF-BM antibody. (**B**) ELISA curves of the secondary antibody against the anti-SF-AP antibody.

It was necessary to establish optimal antibody dilutions to identify the silks. Figure 4 shows the indirect ELISA curves of the secondary antibody against the primary antibody when applied at different dilutions. As shown in Fig. 4A,B, the optical density (OD_450_) values decreased with an increasing dilution of the primary antibody. The anti-SF-BM antibody was diluted 1:500, 1:1000, 1:2000, 1:4000, 1:8000 and 1:16,000, while the anti-SF-AP antibody was diluted 1:1500, 1:3000, 1:6000, 1:12,000, 1:24,000 and 1:48,000 with the same blocking solution. Besides, the secondary antibody was diluted 1:500, 1:1000, 1:2000, 1:4000 and 1:8000 with blocking solution accordingly. To obtain a high signal-to-background ratio and to avoid false-positive results, the antibody dilutions giving a favorable OD_450_ (approximately 1.0) were chosen. Considering the cost and the precision of the result, an anti-SF-BM antibody dilution ratio of 1:1000 and a secondary antibody dilution of 1:1000 were selected as the optimal antibody dilutions. Analogously, dilution ratios of 1:5000 for the anti-SF-AP antibody and 1:2000 for the secondary antibody were chosen. Under the optimal conditions, the OD_450_ value of positive control was approximately 1.0, while the negative control was present in low background levels, ensuring the sensitivity of the following ELISA tests.

#### Cross-reactivity

**Figure 5:**
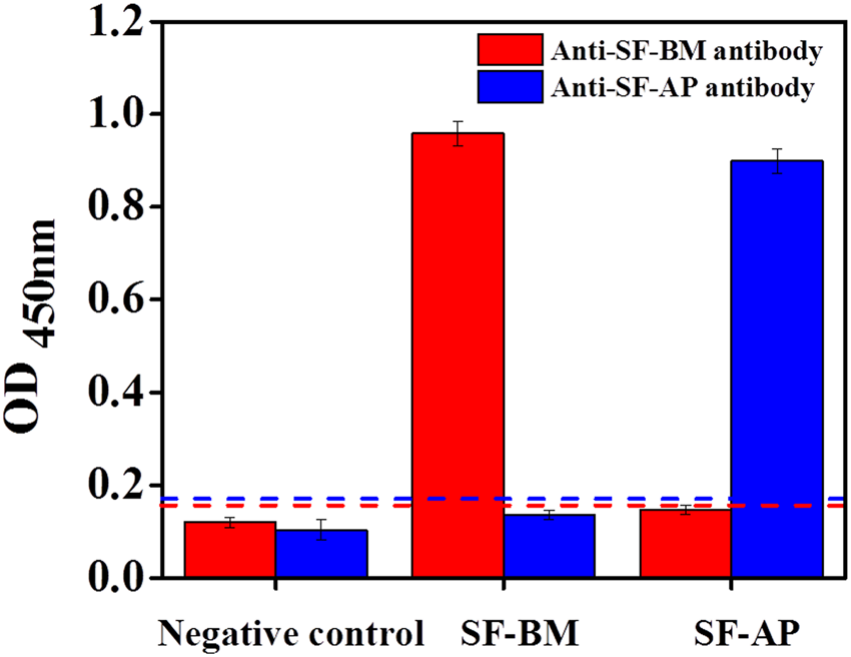
Cross-reactivity of the anti-SF-BM and anti-SF-AP antibodies at optimized antibody dilutions. The two dashed lines indicate the cut-off values.

Figure 5 presents the cross-reactivity results for the *B. mori* and *A. pernyi* silks. Silk fibroin was detected by the anti-SF-BM antibody and the anti-SF-AP antibody under the optimal antibody dilutions. As shown in Fig. 5, the OD_450_ value for the *B. mori* silk detected using the anti-SF-BM antibody was much higher than the cut-off, but the application of the anti-SF-BM antibody to *A. pernyi* silk yielded a negative result in the immunological test. Similarly, the anti-SF-AP antibody detected only *A. pernyi* silk and did not detect *B. mori* silk. Strikingly, there was no cross-reactivity between *B. mori* and *A. pernyi*. These results indicated that the antibodies were prepared well and exhibited high specificity for the two different silk fibroins. Therefore, the developed ELISAs can successfully identify the species that produce each silk.

#### Species identification of silk samples via ELISA

**Figure 6:**
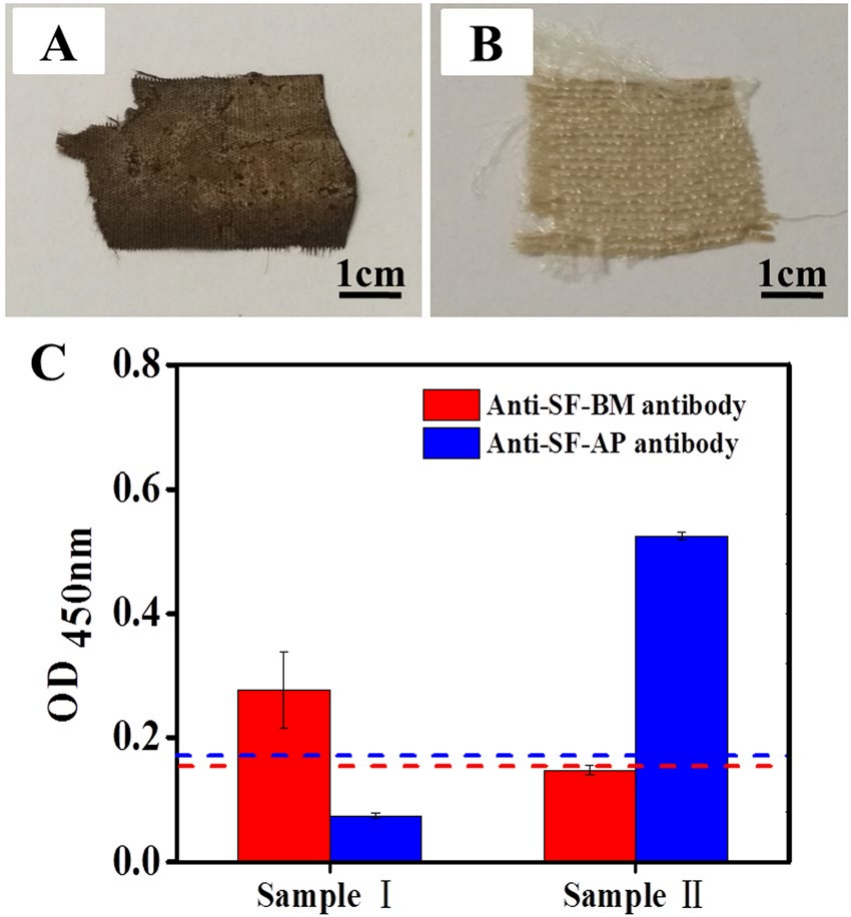
Digital images of Sample I (**A**) and Sample II (**B**). (**C**) ELISA results for Sample I and Sample II. The two dashed lines indicate the cut-off values.

The species that produced the silk samples were subsequently identified via ELISA using the highly specific antibodies under the optimal conditions. Figure 6A,B show images of Sample I and Sample II. Sample I, which was unearthed from a Chu tomb (approximately 2300 years old) in Anji county of Zhejiang province, appeared brown and exhibited a rotted morphology. Sample II which exhibited few impurities and a well-woven construction, was kindly provided by Prof. Feng Zhao in China National Silk Museum.

To evaluate the accuracy of the established ELISA method for given silk samples, the two silks were identified according to the procedures described above. The ELISA results for Sample I and Sample II are shown in Fig. 6C. The anti-SF-BM antibody produced a positive result for Sample I but a negative result for Sample II. In contrast, the OD_450_ value for Sample II with the anti-SF-AP antibody was higher than the cut-off, which indicated that Sample II was produced by *A. pernyi*. Hence, the developed ELISA method with well-designed primary antibodies is practical and valid for identifying which species produced a given silk.

### Species identification of silks via proteomics

#### Biomarkers for B. mori and A. pernyi silk

**Figure 7:**
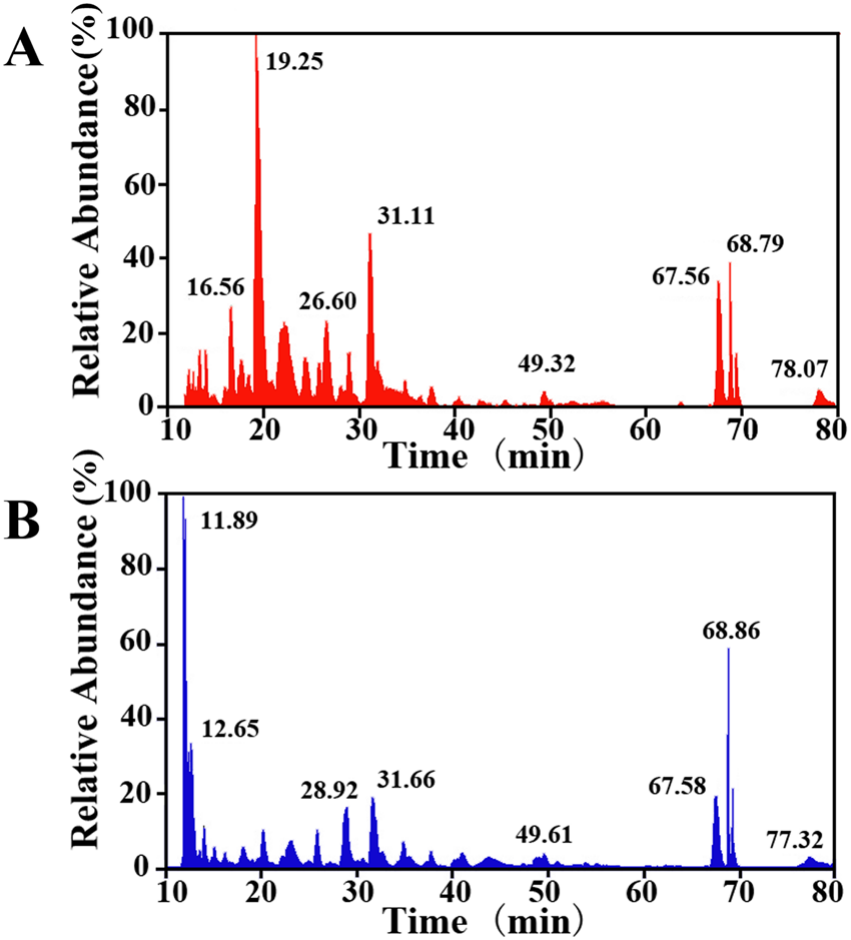
Mass chromatograms of the silk fibroins extracted from *B. mori* silk (**A**) and *A. pernyi* silk (**B**).

The LC-MS/MS chromatograms of the digested fibroins are shown in Fig. 7. The chromatograms represent the most intense peaks of the peptides fragment at every retention time. In Fig. 7, the differences in the two digested fibroins can be easily observed under representative retention times from 10 to 80 min. The prominent peak of the *B. mori* silk appeared at approximately 19.25 min. However, the prominent peak of the *A. pernyi* silk appeared earlier, starting at 11 min and increasing to a maximum at 11.89 min. The two digested fibroins exhibited similar peaks at approximately 68 min, with obvious differences in the peaks occurring at retention times of 10 min to 25 min. The MS/MS chromatograms within this time range were subjected to search using Proteome Discoverer software.

There were thirteen proteins identified and screened in *B. mori* silk, but only four of them showed high score and PSMs. In the *A. pernyi* silk, fifteen proteins were identified, among which only two were credible proteins relevant to fibroin with high score and PSMs. The other proteins in the *B. mori* and *A. pernyi* silk displayed low signals and were not reliably identified. Table 2 shows the seven proteins detected in *B. mori* and *A. pernyi* silk. Fibroin heavy chain and fibroin light chain numbered as P05790 and P21828, respectively, were identified as components of fibroin derived from *B. mori* silk. Fibroin heavy chain fragment (Q99050) and fibrohexamerin (Q9BLL8) originated from *Bombyx mandarina* (*B. mandarina*) were also detected, which could be explained because both *B. mori* and modern *B. mandarina* were originated from ancient *B. mandarina*^40–42^. No proteins ascribed to *A. pernyi* silk fibroin were detected in the *B. mor*i silk. Next, *A. pernyi* silk was also evaluated, and three proteins were identified: fibroin (O76786), fibroin heavy chain (A0A0K0KR73) and fibroin fragment (Q8ISB3). The fibroin (O76786) displayed high score and PSMs, which credibly verified the silk from *A. pernyi*. Interestingly, fibroin heavy chain (A0A0K0KR73) ascribed to *Antheraea assama* and fibroin fragment (Q8ISB3) ascribed to *Antheraea mylitta* were also observed, which may be attributed to the hybridization of *A. pernyi* with *Antheraea assama* and *Antheraea mylitta* genome. And, withal, none of the proteins from *B. mori* silk were present in *A. pernyi* silk. The unique peptides of proteins greater than or equal to 2 are generally considered to be more reliable. Therefore, the four proteins detected in *B. mori* silk and two proteins (O76786, Q8ISB3) detected in *A. pernyi* silk could potentially be used as biomarkers for identifying *Bombyx* and *Antheraea* genera.

**Table 2:** Proteins bearing different species-diagnostic peptides between *B. mori* and *A. Pernyi* silk.

Sample	Accession number^a^	Protein name	Score^b^	Coverage	Matched peptides	Unique Peptides	PSMs^c^	Species
*B. mori* silk	P05790	Fibroin heavy chain	798.51	1.86	14	12	240	*B. mori*
	Q99050	Fibroin heavy chain (fragment)	334.15	24.16	5	3	107	*B. mandarina*
	P21828	Fibroin light chain	229.25	25.95	7	7	110	*B. mori*
	Q9BLL8	Fibrohexamerin	51.76	23.64	7	7	26	*B. mandarina*
*A. pernyi* silk	O76786	Fibroin	2280.07	28.38	26	18	577	*A. Pernyi*
	A0A0K0KR73	Fibroin heavy chain	457.70	3.31	6	1	118	*A. assama*
	Q8ISB3	Fibroin (fragment)	303.42	14.20	5	2	112	*A. mylitta*

^a^Accession number corresponds to the UniProt accession number.

^b^Score obtained from LC-MS/MS identification.

^c^The peptide spectrum matches.

To gain a better understanding of the amino acid sequences of *B. mori* and *A. pernyi* silk, peptides supporting the identification of fibroin in *B. mori* and *A. pernyi* silk are shown in Tables S-1 and S-2, respectively. Altogether, 74 peptide fragments were detected, and eleven peptides fragments were post-translationally modified by the oxidation of methionine or carbamidomethylation of cysteine. A total of 37 peptide fragments were observed in *B. mori* silk, and the remaining peptides were detected in *A. pernyi* silk. Modifications mainly occurred in *B. mori* silk, and there were no modifications detected in *A. pernyi* silk. All peptide fragments digested with trypsin end in arginine or lysine, which indicated the cutting sites of trypsin.

#### Species identification of ancient silks via proteomics

To explore the practicality of using the selected biomarkers in silk samples, the two silks were identified via proteomics. Table 3 shows the proteomic results for Sample I and Sample II. Protein accession numbered Q99050, one of the biomarkers found in *B. mori* silk, was detected in Sample I. The peptide DASGAVIEEEITTK at m/z 1462.73 corresponded to the detected peptide at m/z 1462.73 in *B. mori* silk. Therefore, it is speculated that Sample I was produced by *Bombyx*. The identified peptides of Q99050 in Sample I and *B. mori* silk are shown in Figure S-1. Moreover, proteins accession numbered Q8ISB3 and O76786 were detected in Sample II. Peptides GDGGYGSDSAAAAAAAAAAAAGSGAGGR, at m/z 2208.99, and NAATRpHLSGNER, at m/z 1422.72, of Q8ISB3 were common peptides that were detectable in both Sample II and *A. pernyi* silk. All the ten peptides from O76786 were also identified in *A. pernyi* silk. Figures S-2 and S-3 show the identified peptides of Q8ISB3 and O76786, respectively. Thus, the results revealed that Sample II came from *Antheraea*, which could also be verified via ELISA. Consequently, the biomarkers for *Bombyx* and *Antheraea* obtained through proteomics were shown to be effective in an archaeological application. Furthermore, the proteomics method is useful for identifying silk-producing species and could provide positive proof at the sequence level.

**Table 3:** Proteins bearing species-diagnostic peptides identified in Sample I and Sample II.

Sample	Accessio number	Protein name	Score	Unique peptides	PSMs	MW [kDa]
Sample I	Q99050	Fibroin heavy chain (fragment)	2.65	1	1	18.3
Sample II	O76786	Fibroin	134.04	9	51	215.9
	Q8ISB3	Fibroin (fragment)	277.88	7	90	45.4

## Conclusion

In contrast to traditional techniques, the present study provides a series of novel methods for identifying silks at the species and genus levels. In particular, *B. mori* silk and *A. pernyi* silk were identified and distinguished from each other by combining traditional methods with immunological techniques and proteomics. Significant differences between *B. mori* silk and *A. pernyi* silk were found, ranging from morphology to primary structure. As traditional methods are not applicable for identifying silks with contamination or severe degradation, ELISA and proteomics were applied to distinguish silk samples. The ELISA results showed that the antibodies exhibited high specificity for combining with the corresponding antigen and did not cross-react with the other antigen. Through proteomic analysis, biomarkers for *Bombyx* and *Antheraea* were identified. The subsequent results revealed that the species that produced a given silk sample can be identified using these biomarkers. Thus, ELISA and proteomics are suitable and efficient tools for identifying the species that produced a sample of silk. The strategy that we adopted in this study will lead to new research avenues for archaeological samples.

## Supplementary information

Supporting Information for: Species Identification of Bombyx mori and Antheraea pernyi silk via Immunology and Proteomics
