# Supplementary material for: Species identification of Bombyx mori and Antheraea pernyi silk via immunology and proteomics
Source: Sci Rep. 2019 Jun 28;9:9381. doi: 10.1038/s41598-019-45698-8 (PMC6599025; doi:10.1038/s41598-019-45698-8)
Supplement: Supplementary file 1 — Supporting Information for: Species Identification of Bombyx mori and Antheraea pernyi silk via Immunology and Proteomics [file 41598_2019_45698_MOESM1_ESM.pdf]

Supporting Information for:

Species Identification of *Bombyx mori* and *Antheraea pernyi* silk via Immunology and Proteomics

Jincui Gu[a], Qingqing Li[a], Boyi Chen[a], Chengfeng Xu[a], Hailing Zheng[b], Yang Zhou[b, *], Zhiqin Peng[c], Zhiwen Hu[c], Bing Wang[a,*]

a.Key Laboratory of Advanced Textile Materials and Manufacturing Technology, Ministry of Education, Zhejiang Sci-Tech University, Hangzhou 310018, China

b.Key Scientific Research Base of Textile Conservation, State Administration for Cultural Heritage, China National Silk Museum, Hangzhou 310002, China

c.Institute of Textile Conservation, Zhejiang Sci-Tech University, Hangzhou 310018, China

*Email: wbing388@163.com (B. Wang) and cnsmzhouyang@126.com; Telephone and fax: +86-571-86843867.

Table of contents

Supplementary Tables

Table S-1 Species-diagnostic peptides identified in silk produced by *B. mori*. S-2

Table S-2 Species-diagnostic peptides identified in silk produced by *A. pernyi*. S-4

Supplementary figures

Figure S-1. The sequence of intact Q99050 protein. S-6

Figure S-2. The sequence of partial O76786 protein. S-7

Figure S-3. The sequence of intact Q8ISB3 protein. S-8

Figure S-4. The fragment peaks of peptide DASGAVIEEEITTK identified in both Sample I and *B. mori* silk.

Figure S-5. The fragment peaks of peptides (numbered as O76786) identified in both Sample II and *A. pernyi* silk.

Figure S-6. The fragment peaks of peptides (numbered as Q8ISB3) identified in both Sample II and *A. pernyi* silk.

Table S-1 Species-diagnostic peptides identified in silk produced by *B. mori*.

Accession	Sequence	# PSMs	Modifications	MH+ [Da]	ΔM [ppm]	RT [min]	# Missed Cleavages
P05790	GYGQGAGSAASSVSSASSRSYDYSRR	4		2614.18178	-2.28	13.18	2
	GYGQGAGSAASSVSSASSRSYDYSR	7		2458.08139	-2.13	15.14	1
	GYGQGAGSAASSVSSASSR	14		1686.76389	-2.35	13.26	0
	DASGAVIEEQITTKKmQR	13	M16(Oxidation)	2021.03391	0.31	14.67	2
	TFVITTDSDGNESIVEEDVLmK	37	M21(Oxidation)	2458.16123	2.83	51.08	0
	TFVITTDSDGNESIVEEDVLMK	45		2442.15898	-0.15	60.45	0
	DASGAVIEEQITTKK	33		1589.83513	-1.91	16.22	1
	DASGAVIEEQITTK	31		1461.74077	-1.66	26.86	0
	REGYEYAWSSK	16		1375.62798	0.17	16.10	1
	mIKTFVITTDSDGNESIVEEDVLmK	6	M1(Oxidation); M24(Oxidation)	2846.37906	3.63	48.28	1
	EGYEYAWSSK	25		1219.52605	-0.47	25.31	0
	MIKTFVITTDSDGNESIVEEDVLmK	3	M24(Oxidation)	2830.36997	-1.36	51.36	1
	SYDYSRRNVR	1		1315.64902	-0.90	12.02	2
	RQLVVKFR	1		1045.66399	0.98	13.00	2
	FRALPcVNc	2	C6(Carbamidomethyl); C9(Carbamidomethyl)	1136.53276	-1.09	23.24	1
	ALPcVNc	2	C4(Carbamidomethyl); C7(Carbamidomethyl)	833.36357	-1.06	17.96	0
Q99050	TFVITTDSDGNESIVEEDVLmK	37	M21(Oxidation)	2458.16123	2.83	51.08	0
	TFVITTDSDGNESIVEEDVLMK	45		2442.15898	-0.15	60.45	0
	DASGAVIEEEITTK	5		1462.73003	1.92	30.50	0
	DASGAVIEEEITTKK	6		1590.83530	8.24	23.53	1
	DASGAVIEEEITTKKmQR	5	M16(Oxidation)	2022.02396	3.29	16.49	2
	mIKTFVITTDSDGNESIVEEDVLmK	6	M1(Oxidation); M24(Oxidation)	2846.37906	3.63	48.28	1
	MIKTFVITTDSDGNESIVEEDVLmK	3	M24(Oxidation)	2830.36997	-1.36	51.36	1
P21828	AWDYVDDTDKSIAILNVQEILK	17		2549.32413	4.18	68.66	1

	DIDDGKASSVISR	18		1362.68547	-0.40	15.32	1
	SIAILNVQEILK	12		1340.81487	0.02	56.38	0
	YIAQAASQVHV	47		1186.62029	-1.05	18.08	0
	AWDYVDDTDK	9		1227.51885	1.93	23.66	0
	YSVGPALGcAGGGR	1	C9(Carbamidomethyl)	1321.63384	1.53	18.58	0
	SGNFAGFR	6		855.41100	0.21	16.84	0
Q9BLL8	DLTLLHIYEREHIFGK	5		1984.05996	-2.61	45.36	1
	DLTLLHIYER	8		1272.69402	-0.54	42.00	0
	VSEFYDNVR	7		1128.53008	-1.75	18.50	0
	GQIPSQYEIPVFR	1		1533.80998	2.56	43.97	0
	TAQWLSK	1		833.45098	-0.76	15.16	0
	EHIFGK	1		730.38774	-0.74	12.46	0
	LTTVFDK	3		823.45445	-1.92	17.47	0

Table S-2 Species-diagnostic peptides identified in silk produced by *A. pernyi*.

Accession	Sequence	# PSMs	Modifications	MH+ [Da]	ΔM [ppm]	RT [min]	# Missed Cleavages
O76786	RVPGASSSAAAASSASAGSGQTIIVER	76		2488.27750	0.94	18.24	1
	GGGYGWGDGGYGSDSAAAAAAAAAAAAGSGAGGR	25		2786.21738	0.81	53.85	0
	VPGASSSAAAASSASAGSGQTIIVER	79		2332.17021	-1.64	24.86	0
	SGHDRAYGAGSAAAAAAAAAAGAGASR	11		2287.09487	1.09	25.55	1
	QASHGAGGAAGAAAGAAAGSSAR	7		1824.86269	-3.83	12.31	0
	AAGSAAAAAAAAAAAAASGAGR	21		1585.80722	1.89	32.79	0
	NLRHHDEYVDNHGQLVER	1		2231.06118	-4.01	12.52	1
	AYGAGSAAAAAAAAAAGAGASR	35		1734.85837	3.73	33.30	0
	GDGGYGSGSSAAAAAAAAAASAAR	50		1938.88628	-1.99	37.65	0
	LVETIVLEEDPYGHEDIYEEDVVIKR	20		3102.54824	-1.17	49.23	1
	HHDEYVDNHGQLVERFTTR	8		2353.11025	1.42	14.64	1
	LVETIVLEEDPYGHEDIYEEDVVIK	76		2946.45609	1.81	56.52	0
	GDGGYGSGSSAAAAAAAAAAAAR	59		1851.85600	-1.14	35.11	0
	GDGGYGSGGSSAAAAAAAAAAAR	20		1908.88034	0.40	34.54	0
	GDGGYGSGSSAAAAAAAAAASAARR	7		2094.99588	2.21	33.04	1
	HHDEYVDNHGQLVER	5		1847.83445	-4.09	12.47	0
	GDGGYGSGSSAAAAAAAAAAAAAR	33		1922.89226	-1.55	40.43	0
	GDGGYGSGSSAAAAAAAAAAAARR	15		2007.96036	0.56	30.79	1
	HFERNAATRPHLSGNER	1		1991.97841	-6.20	12.03	1
	GDGGYGSGGSSAAAAAAAAAAAARR	8		2064.98105	0.17	30.46	1
	NAATRPHLSGNER	4		1422.71755	-1.52	11.66	0
	KHFERNAATRPHLSGNER	3		2120.07452	-5.29	11.91	2
	GDGGYGSGSSAAAAAAAAAAAAARR	7		2078.99558	-0.37	35.69	1
	HHDEYVDNHGQLVERFTTRK	1		2481.20390	0.82	13.30	2

	FTTRKHFER	3	1221.64743	-1.09	11.71	2
	GDGGYGSGSSAAAAAAAAAAAAARRAGHDR	2	2544.20957	1.87	25.23	2
A0A0K0KR73	GDGGYGSGSSAAAAAAAAAAAAR	59	1851.85600	-1.14	35.11	0
	GDGGYGSGSSAAAAAAAAAAAAAR	33	1922.89226	-1.55	40.43	0
	GDGGYGSGSSAAAAAAAAAAAAARR	15	2007.96036	0.56	30.79	1
	GDGGYGSGSSAAAAAAAAAAAAAARR	7	2078.99558	-0.37	35.69	1
	FTTRKHFER	3	1221.64743	-1.09	11.71	2
	GGFYESHDSFVVDSSYGSSSSSSAAAAAGSGAGGR	1	3272.42575	7.86	52.90	0
Q8ISB3	LVETIVLEEDPYGHEDIYEEDVVIKR	20	3102.54824	-1.17	49.23	1
	LVETIVLEEDPYGHEDIYEEDVVIK	76	2946.45609	1.81	56.52	0
	GDGGYGSDSAAAAAAAAAAAAAGSGAGGR	11	2208.98760	0.46	41.71	0
	NAATRPHLSGNER	4	1422.71755	-1.52	11.66	0
	KHYERNAATRPHLSGNER	1	2136.06215	-8.65	11.90	2

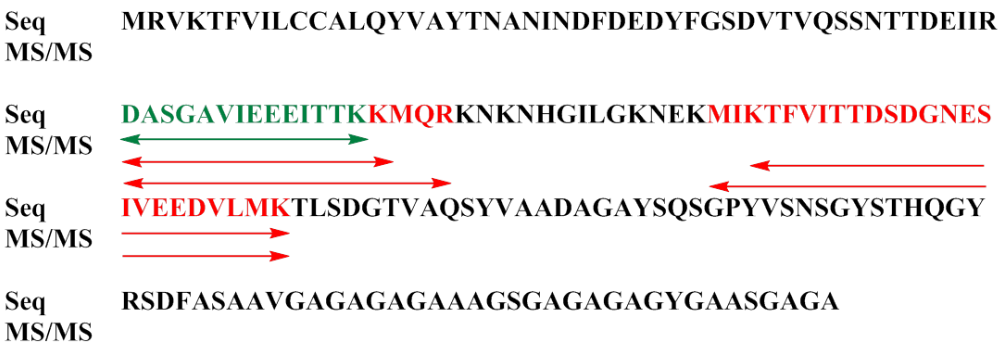

Figure S-1. The sequence of intact Q99050 protein. Peptides identified by LC-MS/MS were underlined. The red lines indicate peptides only identified in *B. mori* silk, while the green lines indicate peptides identified in both Sample I and *B. mori* silk.

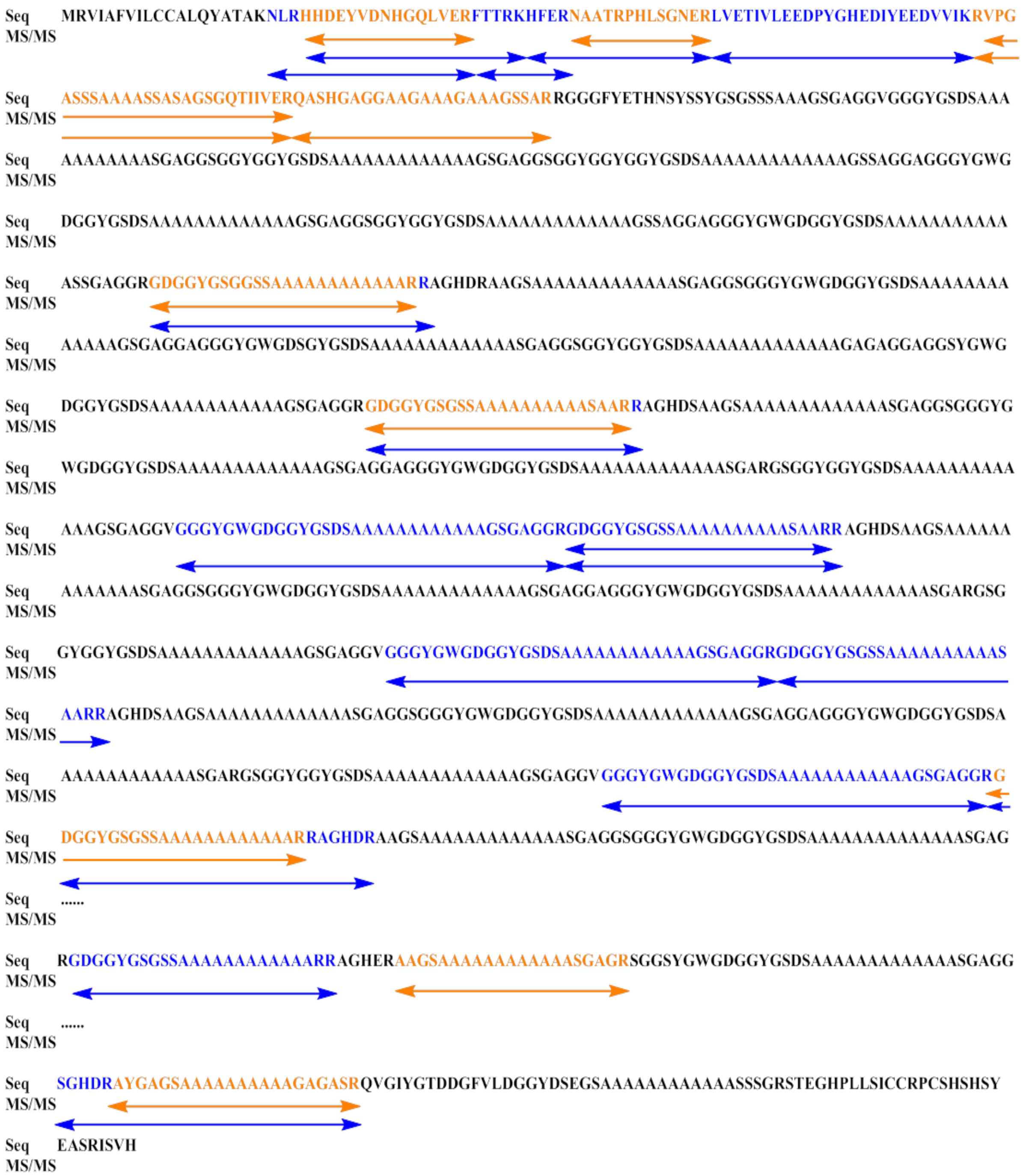

Figure S-2. The sequence of partial O76786 protein. Peptides identified by LC-MS/MS were underlined. The blue lines indicate peptides only identified in *A. pernyi* silk, while the orange lines indicate peptides identified in both Sample II and *A. pernyi* silk.

Seq MRVIAFVILCCALQYATAKNIHHDEYVDSHGQLVERFTTRKHYERNAATRPHLSGNERLVETIVLEEDPYGHEDIYEEDVVIKRVPGA
MS/MS

Seq SSSAAAASSASAGSGQTITVERQASHGAGGAAGAAAGAAASSSVRGGGGFYETHDSYSSYGSDSAAAAAAAAASGAGGRGHGGYGSD
MS/MS

Seq SAAAA AAAAAAAAAAASGAGGRGHGGYGSDSAAAAAAAAAAAAAAAGSGAGGRGDGGYGWDGGYGSDSGAAAAAAAAAAAAAS
MS/MS

Seq GAGGRGDGGYGRGDGGYGSDSAAAAAAAAAAAAAGSGAGGQATVVMDGAMAAMVLTRAQQQLAAAAAAASASGAGGSGGSYEWD
MS/MS

Seq DYGSYGSDSAAAAAAAAAAAAAAAGSGAGGVGGGYGRGDGGYGSDSAAAAAAAAAAAAAGSGAGGRGDGGYGWDGGYGSDSGAAAA
MS/MS

Seq AAAAAAAAASGAGGRGDGGYGWDGGYGSDPGAAAAAAAAAAAAAASGARGRGDGGYGSGSSAAAAAAAAAAAASAARRAGHDRA
MS/MS

Figure S-3. The sequence of intact Q8ISB3 protein. Peptides identified by LC-MS/MS were underlined. The blue lines indicate peptides only identified in *A. pernyi* silk, while the orange lines indicate peptides identified in both Sample II and *A. pernyi* silk

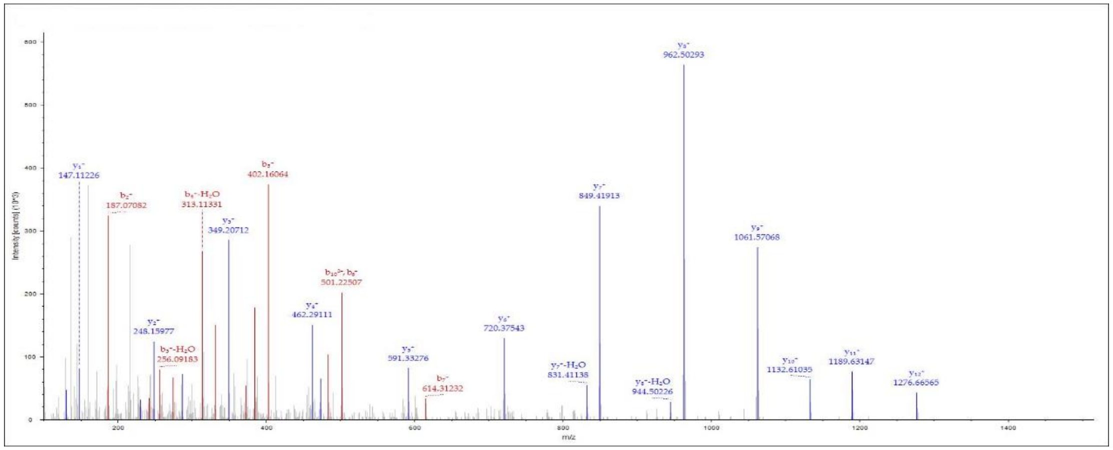

Figure S-4 The fragment peaks of peptide DASGAVIEEEITTK.

#1	b-H_2O^+	b-H_2O^{2+}	Seq.	y-H_2O^+	y-H_2O^{2+}	y-NH_3^+	y-NH_3^{2+}	#2
1	98.02366	49.51547	D					14
2	169.06078	85.03403	A	1329.68971	665.34849	1330.67372	665.84050	13
3	256.09281	128.55004	S	1258.65259	629.82993	1259.63660	630.32194	12
4	313.11428	157.06078	G	1171.62056	586.31392	1172.60457	586.80592	11
5	384.15140	192.57934	A	1114.59909	557.80318	1115.58310	558.29519	10
6	483.21982	242.11355	V	1043.56197	522.28462	1044.54598	522.77663	9
7	596.30389	298.65558	I	944.49355	472.75041	945.47756	473.24242	8
8	725.34649	363.17688	E	831.40948	416.20838	832.39349	416.70038	7
9	854.38909	427.69818	E	702.36688	351.68708	703.35089	352.17908	6
10	983.43169	492.21948	E	573.32428	287.16578	574.30829	287.65778	5
11	1096.51576	548.76152	I	444.28168	222.64448	445.26569	223.13648	4
12	1197.56344	599.28536	T	331.19761	166.10244	332.18162	166.59445	3
13	1298.61112	649.80920	T	230.14993	115.57860	231.13394	116.07061	2
14			K			130.08626	65.54677	1

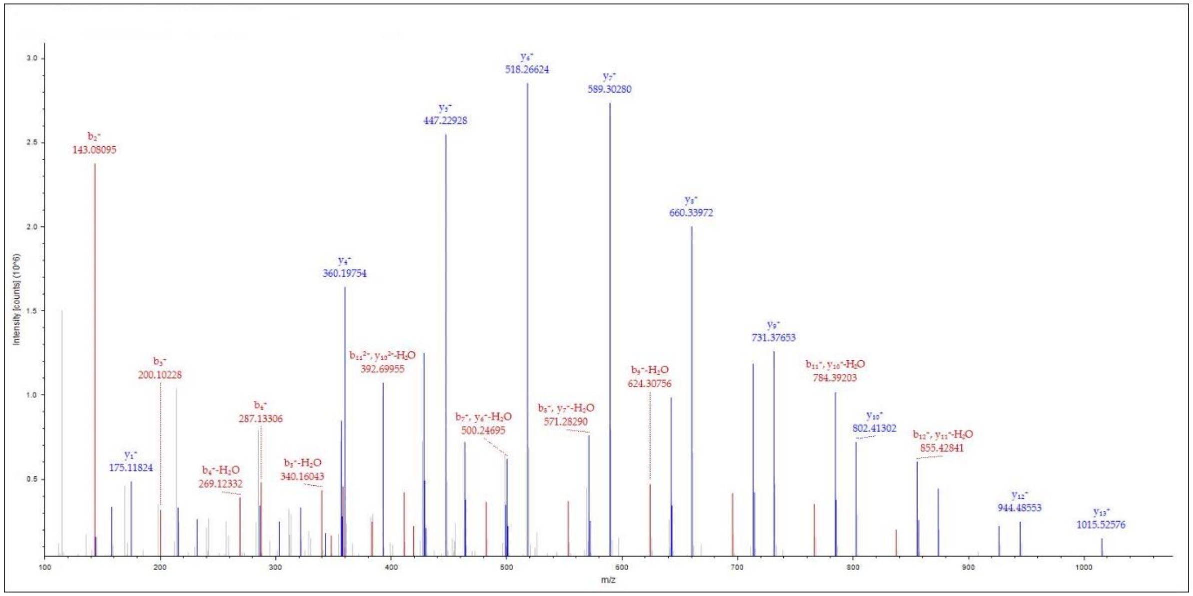

Figure S-5A The fragment peaks of peptide AAGSAAAAAAAAAAAASGAGR.

#1	b-H_2O^+	b-H_2O^{2+}	b-H_2O^{3+}	Seq.	y-H_2O^+	y-H_2O^{2+}	y-H_2O^{3+}	y-NH_3^+	y-NH_3^{2+}	y-NH_3^{3+}	#2
1				A							21
2				A	1496.75655	748.88191	499.59037	1497.74056	749.37392	499.91837	20
3				G	1425.71943	713.36335	475.91133	1426.70344	713.85536	476.23933	19
4	269.12445	135.06586	90.37967	S	1368.69796	684.85262	456.90417	1369.68197	685.34462	457.23218	18
5	340.16157	170.58442	114.05871	A	1281.66593	641.33660	427.89349	1282.64994	641.82861	428.22150	17
6	411.19869	206.10298	137.73775	A	1210.62881	605.81804	404.21445	1211.61282	606.31005	404.54246	16
7	482.23581	241.62154	161.41679	A	1139.59169	570.29948	380.53541	1140.57570	570.79149	380.86342	15
8	553.27293	277.14010	185.09583	A	1068.55457	534.78092	356.85637	1069.53858	535.27293	357.18438	14
9	624.31005	312.65866	208.77487	A	997.51745	499.26236	333.17733	998.50146	499.75437	333.50534	13

10	695.34717	348.17722	232.45391	A	926.48033	463.74380	309.49829	927.46434	464.23581	309.82630	12
11	766.38429	383.69578	256.13295	A	855.44321	428.22524	285.81925	856.42722	428.71725	286.14726	11
12	837.42141	419.21434	279.81199	A	784.40609	392.70668	262.14021	785.39010	393.19869	262.46822	10
13	908.45853	454.73290	303.49103	A	713.36897	357.18812	238.46117	714.35298	357.68013	238.78918	9
14	979.49565	490.25146	327.17007	A	642.33185	321.66956	214.78213	643.31586	322.16157	215.11014	8
15	1050.53277	525.77002	350.84911	A	571.29473	286.15100	191.10309	572.27874	286.64301	191.43110	7
16	1121.56989	561.28858	374.52815	A	500.25761	250.63244	167.42405	501.24162	251.12445	167.75206	6
17	1208.60192	604.80460	403.53883	S	429.22049	215.11388	143.74501	430.20450	215.60589	144.07302	5
18	1265.62339	633.31533	422.54598	G				343.17247	172.08987	115.06234	4
19	1336.66051	668.83389	446.22502	A				286.15100	143.57914	96.05519	3
20	1393.68198	697.34463	465.23218	G				215.11388	108.06058	72.37615	2
21				R				158.09241	79.54984	53.36899	1

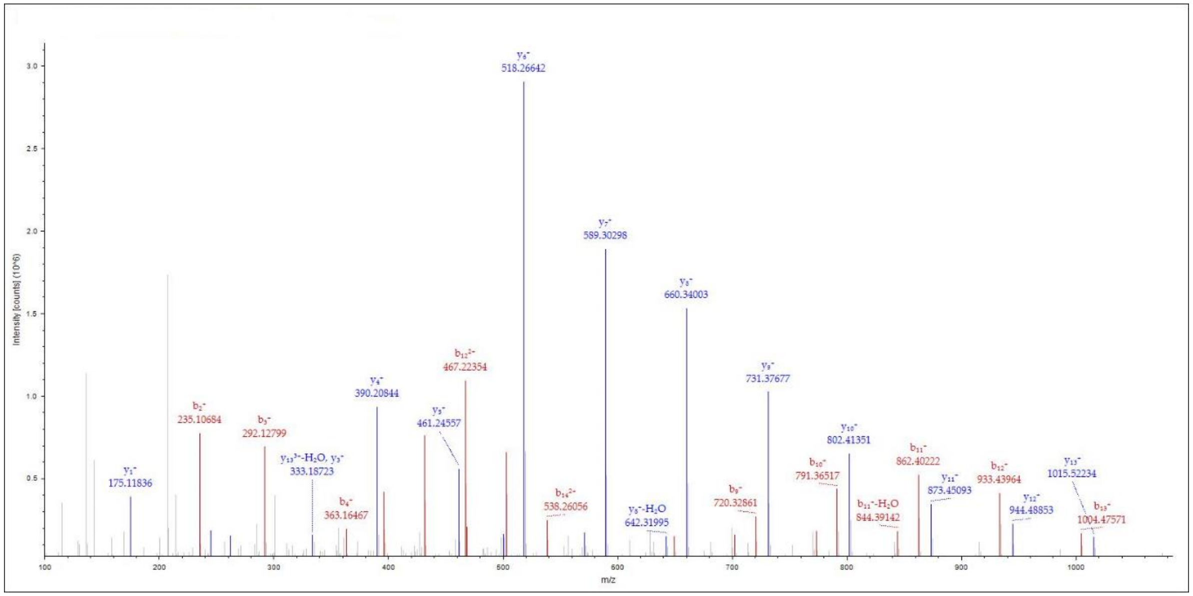

Figure S-5B The fragment peaks of peptide AYGAGSAAAAAAAAAAGAGASR.

#1	b-H_2O^+	b-H_2O^{2+}	b-H_2O^{3+}	Seq.	y-H_2O^+	y-H_2O^{2+}	y-H_2O^{3+}	y-NH_3^+	y-NH_3^{2+}	y-NH_3^{3+}	#2
1				A							22
2				Y	1645.80422	823.40575	549.27292	1646.78823	823.89775	549.60093	21
3				G	1482.74090	741.87409	494.91848	1483.72491	742.36609	495.24649	20
4				A	1425.71943	713.36335	475.91133	1426.70344	713.85536	476.23933	19
5				G	1354.68231	677.84479	452.23229	1355.66632	678.33680	452.56029	18
6	489.20924	245.10826	163.74127	S	1297.66084	649.33406	433.22513	1298.64485	649.82606	433.55314	17
7	560.24636	280.62682	187.42031	A	1210.62881	605.81804	404.21445	1211.61282	606.31005	404.54246	16
8	631.28348	316.14538	211.09935	A	1139.59169	570.29948	380.53541	1140.57570	570.79149	380.86342	15
9	702.32060	351.66394	234.77839	A	1068.55457	534.78092	356.85637	1069.53858	535.27293	357.18438	14
10	773.35772	387.18250	258.45743	A	997.51745	499.26236	333.17733	998.50146	499.75437	333.50534	13

11	844.39484	422.70106	282.13647	A	926.48033	463.74380	309.49829	927.46434	464.23581	309.82630	12
12	915.43196	458.21962	305.81551	A	855.44321	428.22524	285.81925	856.42722	428.71725	286.14726	11
13	986.46908	493.73818	329.49455	A	784.40609	392.70668	262.14021	785.39010	393.19869	262.46822	10
14	1057.50620	529.25674	353.17359	A	713.36897	357.18812	238.46117	714.35298	357.68013	238.78918	9
15	1128.54332	564.77530	376.85263	A	642.33185	321.66956	214.78213	643.31586	322.16157	215.11014	8
16	1199.58044	600.29386	400.53167	A	571.29473	286.15100	191.10309	572.27874	286.64301	191.43110	7
17	1256.60191	628.80459	419.53882	G	500.25761	250.63244	167.42405	501.24162	251.12445	167.75206	6
18	1327.63903	664.32315	443.21786	A	443.23614	222.12171	148.41690	444.22015	222.61371	148.74490	5
19	1384.66050	692.83389	462.22502	G	372.19902	186.60315	124.73786	373.18303	187.09515	125.06586	4
20	1455.69762	728.35245	485.90406	A	315.17755	158.09241	105.73070	316.16156	158.58442	106.05871	3
21	1542.72965	771.86846	514.91474	S	244.14043	122.57385	82.05166	245.12444	123.06586	82.37967	2
22				R				158.09241	79.54984	53.36899	1

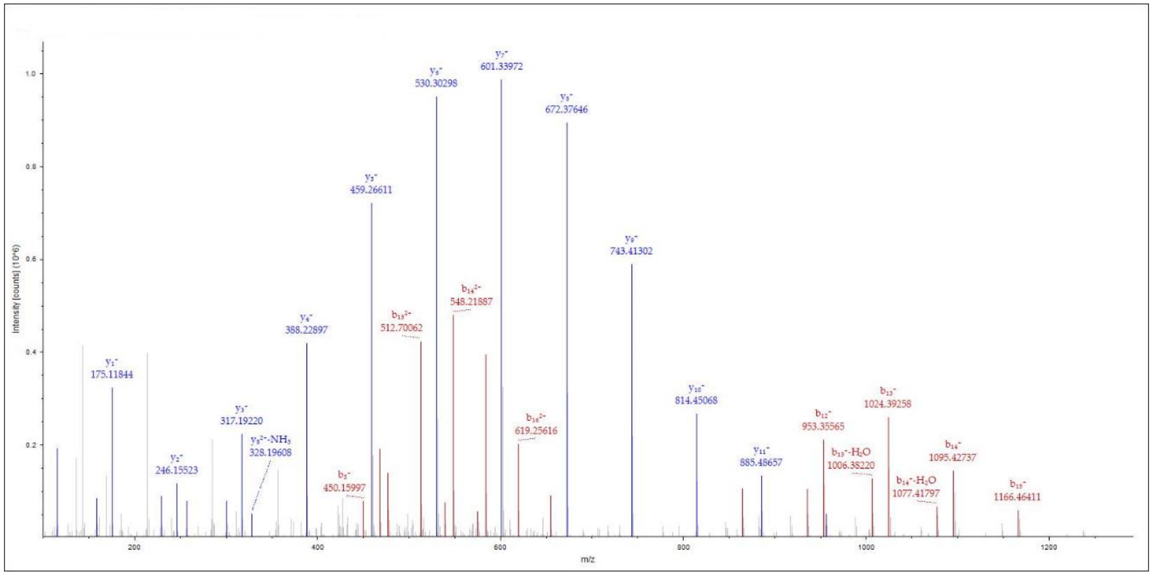

Figure S-5C The fragment peaks of peptide GDGGYGSGGSSAAAAAAAAAAAAR.

#1	b-H_2O^+	b-H_2O^{2+}	b-H_2O^{3+}	Seq.	y-H_2O^+	y-H_2O^{2+}	y-H_2O^{3+}	y-NH_3^+	y-NH_3^{2+}	y-NH_3^{3+}	#2
1				G							24
2	155.04513	78.02620	52.35323	D	1833.84755	917.42741	611.95403	1834.83156	917.91942	612.28204	23
3	212.06660	106.53694	71.36039	G	1718.82060	859.91394	573.61172	1719.80461	860.40594	573.93972	22
4	269.08807	135.04767	90.36754	G	1661.79913	831.40320	554.60456	1662.78314	831.89521	554.93257	21
5	432.15139	216.57933	144.72198	Y	1604.77766	802.89247	535.59740	1605.76167	803.38447	535.92541	20
6	489.17286	245.09007	163.72914	G	1441.71434	721.36081	481.24296	1442.69835	721.85281	481.57097	19
7	576.20489	288.60608	192.73982	S	1384.69287	692.85007	462.23581	1385.67688	693.34208	462.56381	18
8	633.22636	317.11682	211.74697	G	1297.66084	649.33406	433.22513	1298.64485	649.82606	433.55314	17
9	690.24783	345.62755	230.75413	G	1240.63937	620.82332	414.21797	1241.62338	621.31533	414.54598	16
10	777.27986	389.14357	259.76481	S	1183.61790	592.31259	395.21082	1184.60191	592.80459	395.53882	15
11	864.31189	432.65958	288.77548	S	1096.58587	548.79657	366.20014	1097.56988	549.28858	366.52815	14

12	935.34901	468.17814	312.45452	A				1010.53785	505.77256	337.51747	13
13	1006.38613	503.69670	336.13356	A				939.50073	470.25400	313.83843	12
14	1077.42325	539.21526	359.81260	A				868.46361	434.73544	290.15939	11
15	1148.46037	574.73382	383.49164	A				797.42649	399.21688	266.48035	10
16	1219.49749	610.25238	407.17068	A				726.38937	363.69832	242.80131	9
17	1290.53461	645.77094	430.84972	A				655.35225	328.17976	219.12227	8
18	1361.57173	681.28950	454.52876	A				584.31513	292.66120	195.44323	7
19	1432.60885	716.80806	478.20780	A				513.27801	257.14264	171.76419	6
20	1503.64597	752.32662	501.88684	A				442.24089	221.62408	148.08515	5
21	1574.68309	787.84518	525.56588	A				371.20377	186.10552	124.40611	4
22	1645.72021	823.36374	549.24492	A				300.16665	150.58696	100.72707	3
23	1716.75733	858.88230	572.92396	A				229.12953	115.06840	77.04803	2
24				R				158.09241	79.54984	53.36899	1

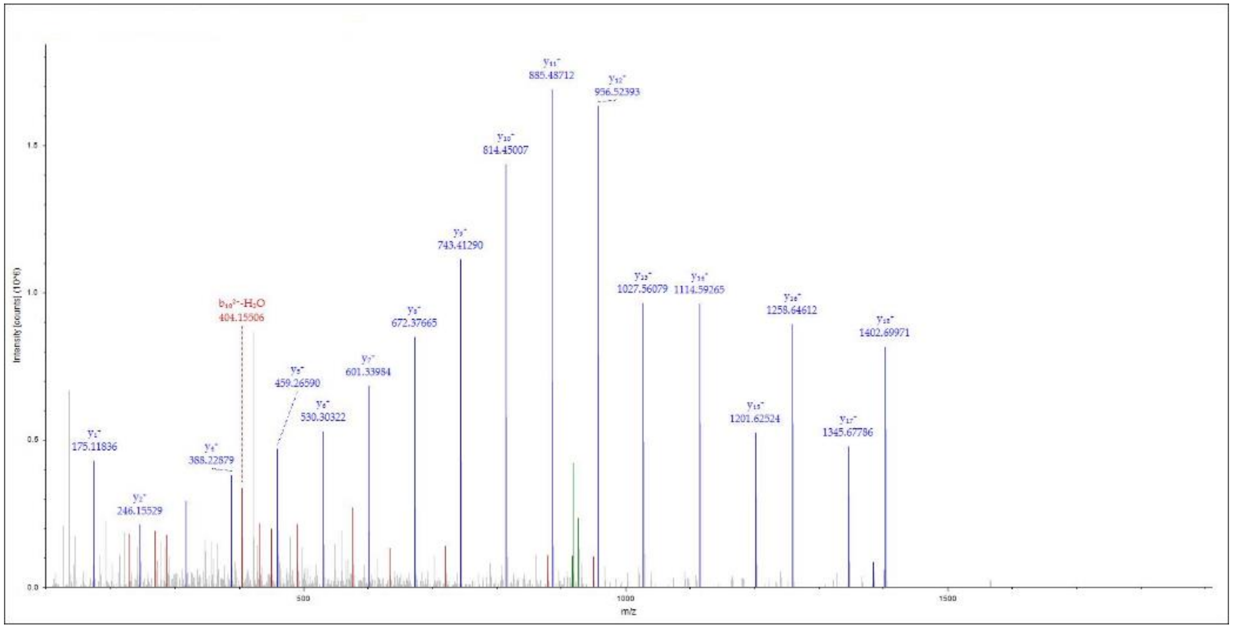

Figure S-5D The fragment peaks of peptide GDGGYGSGSSAAAAAAAAAAAAR.

#1	**b-H_2O^+**	**b-H_2O^{2+}**	**Seq.**	**y-H_2O^+**	**y-H_2O^{2+}**	**y-NH_3^+**	**y-NH_3^{2+}**	#2
1			G					23
2	155.04513	78.02620	D	1776.82608	888.91668	1777.81009	889.40868	22
3	212.06660	106.53694	G	1661.79913	831.40320	1662.78314	831.89521	21
4	269.08807	135.04767	G	1604.77766	802.89247	1605.76167	803.38447	20
5	432.15139	216.57933	Y	1547.75619	774.38173	1548.74020	774.87374	19
6	489.17286	245.09007	G	1384.69287	692.85007	1385.67688	693.34208	18
7	576.20489	288.60608	S	1327.67140	664.33934	1328.65541	664.83134	17
8	633.22636	317.11682	G	1240.63937	620.82332	1241.62338	621.31533	16
9	720.25839	360.63283	S	1183.61790	592.31259	1184.60191	592.80459	15
10	807.29042	404.14885	S	1096.58587	548.79657	1097.56988	549.28858	14

11	878.32754	439.66741	A			1010.53785	505.77256	13
12	949.36466	475.18597	A			939.50073	470.25400	12
13	1020.40178	510.70453	A			868.46361	434.73544	11
14	1091.43890	546.22309	A			797.42649	399.21688	10
15	1162.47602	581.74165	A			726.38937	363.69832	9
16	1233.51314	617.26021	A			655.35225	328.17976	8
17	1304.55026	652.77877	A			584.31513	292.66120	7
18	1375.58738	688.29733	A			513.27801	257.14264	6
19	1446.62450	723.81589	A			442.24089	221.62408	5
20	1517.66162	759.33445	A			371.20377	186.10552	4
21	1588.69874	794.85301	A			300.16665	150.58696	3
22	1659.73586	830.37157	A			229.12953	115.06840	2
23			R			158.09241	79.54984	1

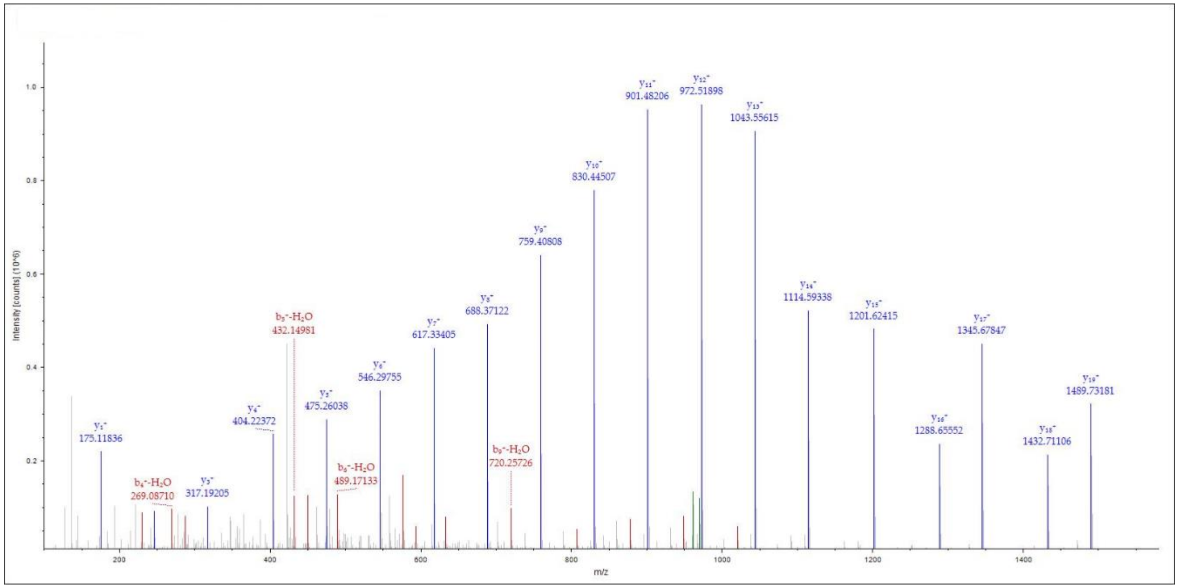

Figure S-5E The fragment peaks of peptide GDGGYGSGSSAAAAAAAAAASAAR.

#1	b-H_2O^+	b-H_2O^{2+}	Seq.	y-H_2O^+	y-H_2O^{2+}	y-NH_3^+	y-NH_3^{2+}	#2
1			G					24
2	155.04513	78.02620	D	1863.85811	932.43269	1864.84212	932.92470	23
3	212.06660	106.53694	G	1748.83116	874.91922	1749.81517	875.41122	22
4	269.08807	135.04767	G	1691.80969	846.40848	1692.79370	846.90049	21
5	432.15139	216.57933	Y	1634.78822	817.89775	1635.77223	818.38975	20
6	489.17286	245.09007	G	1471.72490	736.36609	1472.70891	736.85809	19
7	576.20489	288.60608	S	1414.70343	707.85535	1415.68744	708.34736	18
8	633.22636	317.11682	G	1327.67140	664.33934	1328.65541	664.83134	17
9	720.25839	360.63283	S	1270.64993	635.82860	1271.63394	636.32061	16
10	807.29042	404.14885	S	1183.61790	592.31259	1184.60191	592.80459	15
11	878.32754	439.66741	A	1096.58587	548.79657	1097.56988	549.28858	14

12	949.36466	475.18597	A	1025.54875	513.27801	1026.53276	513.77002	13
13	1020.40178	510.70453	A	954.51163	477.75945	955.49564	478.25146	12
14	1091.43890	546.22309	A	883.47451	442.24089	884.45852	442.73290	11
15	1162.47602	581.74165	A	812.43739	406.72233	813.42140	407.21434	10
16	1233.51314	617.26021	A	741.40027	371.20377	742.38428	371.69578	9
17	1304.55026	652.77877	A	670.36315	335.68521	671.34716	336.17722	8
18	1375.58738	688.29733	A	599.32603	300.16665	600.31004	300.65866	7
19	1446.62450	723.81589	A	528.28891	264.64809	529.27292	265.14010	6
20	1517.66162	759.33445	A	457.25179	229.12953	458.23580	229.62154	5
21	1604.69365	802.85046	S	386.21467	193.61097	387.19868	194.10298	4
22	1675.73077	838.36902	A			300.16665	150.58696	3
23	1746.76789	873.88758	A			229.12953	115.06840	2
24			R			158.09241	79.54984	1

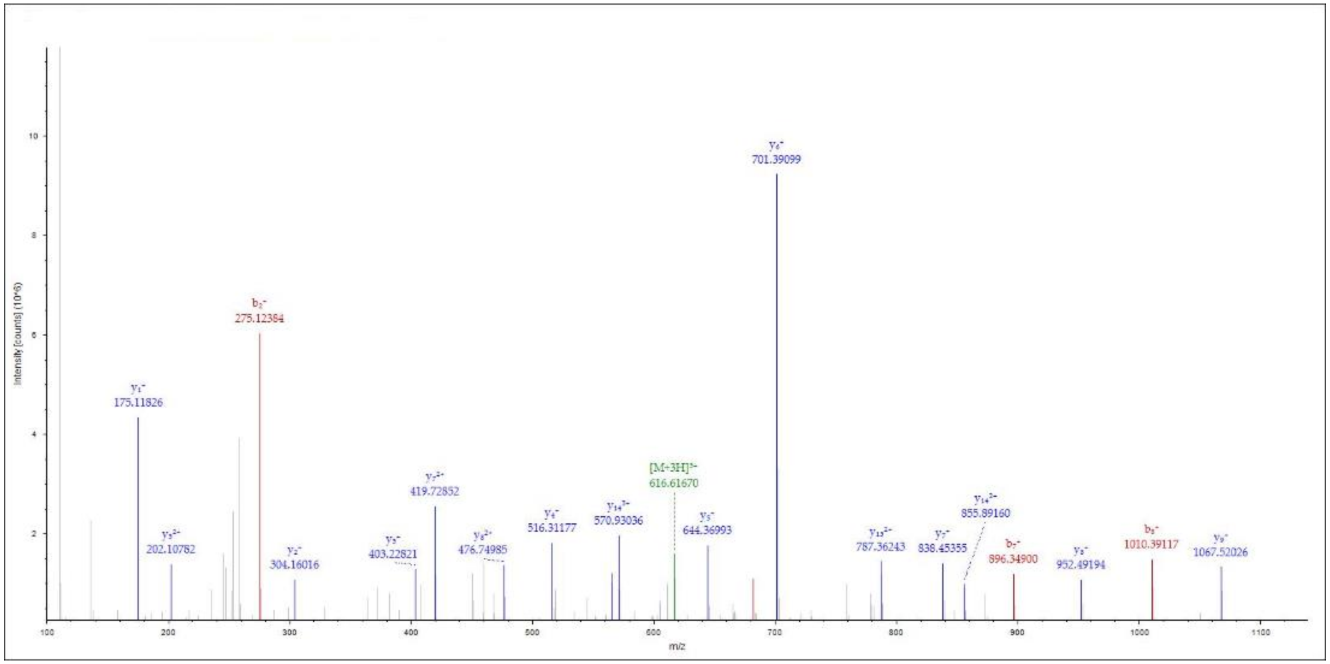

Figure S-5F The fragment peaks of peptide HHDEYVDNHGQLVER.

#1	b-H_2O^+	b-H_2O^{2+}	b-H_2O^{3+}	b-NH_3^+	b-NH_3^{2+}	b-NH_3^{3+}	Seq.	y-H_2O^+	y-H_2O^{2+}	y-H_2O^{3+}	y-NH_3^+	y-NH_3^{2+}	y-NH_3^{3+}	#2
1							H							15
2							H	1692.77253	846.88990	564.92903	1693.75654	847.38191	565.25703	14
3	372.14148	186.57438	124.71868				D	1555.71362	778.36045	519.24272	1556.69763	778.85245	519.57073	13
4	501.18408	251.09568	167.73288				E	1440.68667	720.84697	480.90041	1441.67068	721.33898	481.22841	12
5	664.247	332.627	222.087				Y	1311.644	656.325	437.886	1312.62	656.817	438.214	1

	40	34	32					07	67	21	808	68	21	1
6	763.31582	382.16155	255.11013				V	1148.58075	574.79401	383.53177	1149.56476	575.28602	383.85977	10
7	878.34277	439.67502	293.45244				D	1049.51233	525.25980	350.50896	1050.49634	525.75181	350.83697	9
8	992.38570	496.69649	331.46675	993.36972	497.18850	331.79476	N	934.48538	467.74633	312.16664	935.46939	468.23833	312.49465	8
9	1129.44461	565.22594	377.15306	1130.42863	565.71795	377.48106	H	820.44245	410.72486	274.15233	821.42646	411.21687	274.48034	7
10	1186.46608	593.73668	396.16021	1187.45010	594.22869	396.48822	G	683.38354	342.19541	228.46603	684.36755	342.68741	228.79404	6
11	1314.52466	657.76597	438.84641	1315.50868	658.25798	439.17441	Q	626.36207	313.68467	209.45887	627.34608	314.17668	209.78688	5
12	1427.60873	714.30800	476.54110	1428.59275	714.80001	476.86910	L	498.30349	249.65538	166.77268	499.28750	250.14739	167.10069	4
13	1526.67715	763.84221	509.56390	1527.66117	764.33422	509.89191	V	385.21942	193.11335	129.07799	386.20343	193.60535	129.40600	3
14	1655.71975	828.36351	552.57810	1656.70377	828.85552	552.90611	E	286.15100	143.57914	96.05518	287.13501	144.07114	96.38319	2
15							R				158.09241	79.54984	53.36899	1

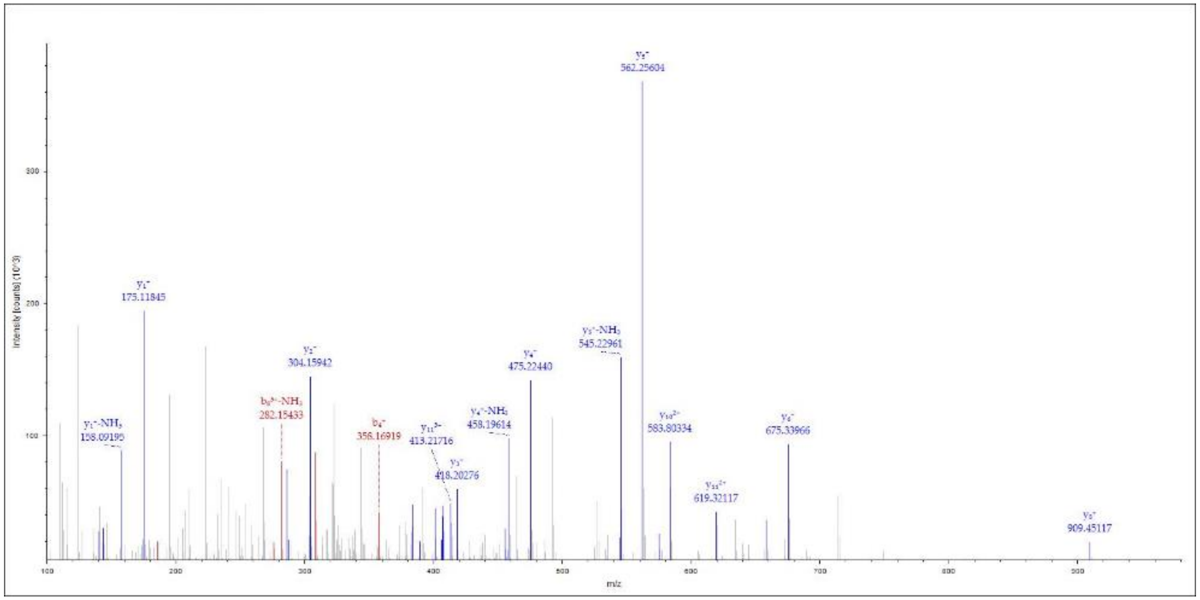

Figure S-5G The fragment peaks of peptide NAATRPHLSGNER.

#1	b-H_2O^+	b-H_2O^{2+}	b-H_2O^{3+}	b-H_2O^{4+}	b-NH_3^+	b-NH_3^{2+}	b-NH_3^{3+}	b-NH_3^{4+}	Seq.	y-H_2O^+	y-H_2O^{2+}	y-H_2O^{3+}	y-H_2O^{4+}	y-NH_3^+	y-NH_3^{2+}	y-NH_3^{3+}	y-NH_3^{4+}	#2
1					98.02366	49.51547	33.34607	25.26137	N									13
2					169.06078	85.03403	57.02511	43.02065	A	1290.66622	645.83675	430.89359	323.42201	1291.65023	646.32875	431.22160	323.66802	12
3					240.09790	120.55259	80.70415	60.77993	A	1219.62910	610.31819	407.21455	305.66273	1220.61311	610.81019	407.54256	305.90874	11
4	340.16156	170.58442	114.05871	85.79585	341.14558	171.07643	114.38671	86.04185	T	1148.59198	574.79963	383.53551	287.90345	1149.57599	575.29163	383.86352	288.14946	10
5	496.2	248.6	166.0	124.8	497.2	249.1	166.4	125.0	R	1047.	524.2	349.8	262.6	1048.	524.7	350.1	262.8	9

	6268	3498	9241	2113	4670	2699	2042	6713		54430	7579	5295	4153	52831	6779	8096	8754	
6	593.31545	297.16136	198.44334	149.08432	594.29947	297.65337	198.77134	149.33032	P	891.44318	446.22523	297.81924	223.61625	892.42719	446.71723	298.14725	223.86226	8
7	730.37436	365.69082	244.12964	183.34905	731.35838	366.18283	244.45764	183.59505	H	794.39041	397.69884	265.46832	199.35306	795.37442	398.19085	265.79633	199.59906	7
8	843.45843	422.23285	281.82433	211.62007	844.44245	422.72486	282.15233	211.86607	L	657.33150	329.16939	219.78202	165.08833	658.31551	329.66139	220.11002	165.33434	6
9	930.49046	465.74887	310.83501	233.37807	931.47448	466.24088	311.16301	233.62408	S	544.24743	272.62735	182.08733	136.81731	545.23144	273.11936	182.41533	137.06332	5
10	987.51193	494.25960	329.84216	247.63344	988.49595	494.75161	330.17017	247.87944	G	457.21540	229.11134	153.07665	115.05931	458.19941	229.60334	153.40466	115.30531	4
11	1101.55486	551.28107	367.85647	276.14417	1102.53888	551.77308	368.18448	276.39018	N	400.19393	200.60060	134.06949	100.80394	401.17794	201.09261	134.39750	101.04994	3
12	1230.59746	615.80237	410.87067	308.40482	1231.58148	616.29438	411.19868	308.65083	E	286.15100	143.57914	96.05518	72.29321	287.13501	144.07114	96.38319	72.53921	2
13									R					158.09241	79.54984	53.36899	40.27856	1

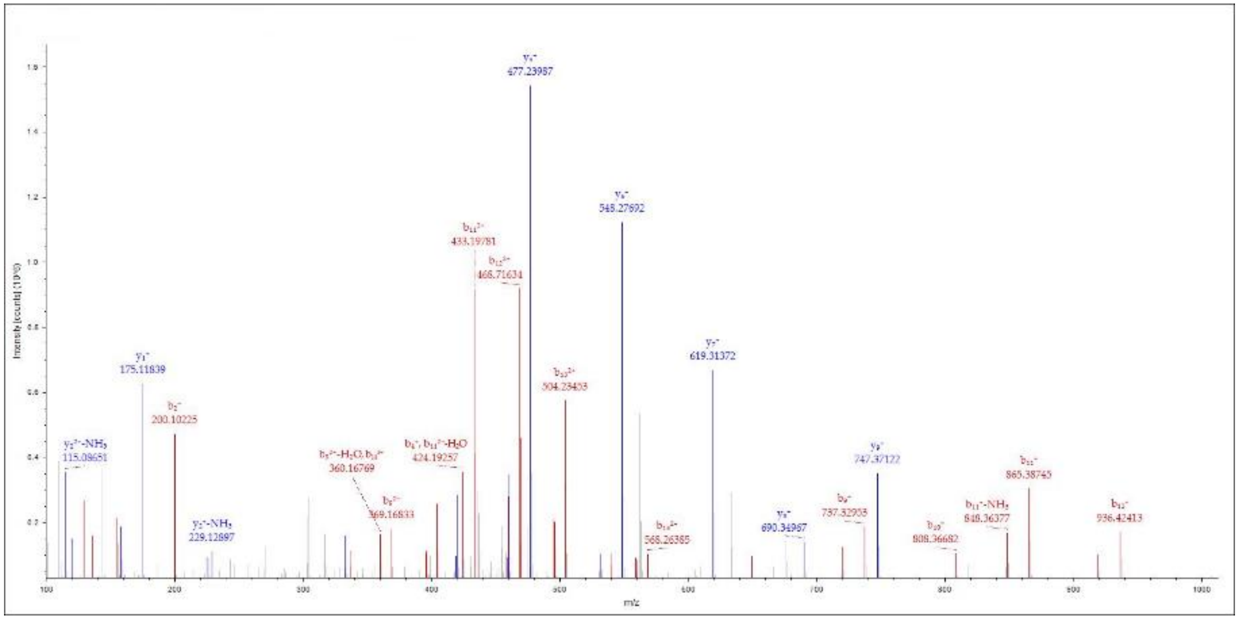

Figure S-5H The fragment peaks of peptide QASHGAGGAAGAAAGAAAGSSAR.

#1	b-H_2O^+	b-H_2O^{2+}	b-H_2O^{3+}	b-H_2O^{4+}	b-NH_3^+	b-NH_3^{2+}	b-NH_3^{3+}	b-NH_3^{4+}	Seq.	y-H_2O^+	y-H_2O^{2+}	y-H_2O^{3+}	y-H_2O^{4+}	y-NH_3^+	y-NH_3^{2+}	y-NH_3^{3+}	y-NH_3^{4+}	#2
1					112.03931	56.52329	38.01795	28.76528	Q									23
2					183.07643	92.04185	61.69699	46.52456	A	1678.80054	839.90391	560.27170	420.45559	1679.78455	840.39591	560.59970	420.70160	22
3	269.12444	135.06586	90.37967	68.03657	270.10846	135.55787	90.70767	68.28257	S	1607.76342	804.38535	536.59266	402.69631	1608.74743	804.87735	536.92066	402.94232	21
4	406.18335	203.59531	136.06597	102.30130	407.16737	204.08732	136.39397	102.54730	H	1520.73139	760.86933	507.58198	380.93830	1521.71540	761.36134	507.90999	381.18431	20
5	463.2	232.1	155.0	116.5	464.1	232.5	155.4	116.8	G	1383.	692.3	461.8	346.6	1384.	692.8	462.2	346.9	1

	0482	0605	7313	5666	8884	9806	0113	0267		67248	3988	9568	7358	65649	3188	2368	1958	9
6	534.24194	267.62461	178.75217	134.31594	535.22596	268.11662	179.08017	134.56195	A	1326.65101	663.82914	442.88852	332.41821	1327.63502	664.32115	443.21653	332.66421	18
7	591.26341	296.13534	197.75932	148.57131	592.24743	296.62735	198.08733	148.81731	G	1255.61389	628.31058	419.20948	314.65893	1256.59790	628.80259	419.53749	314.90493	17
8	648.28488	324.64608	216.76648	162.82668	649.26890	325.13809	217.09448	163.07268	G	1198.59242	599.79985	400.20232	300.40356	1199.57643	600.29185	400.53033	300.64957	16
9	719.32200	360.16464	240.44552	180.58596	720.30602	360.65665	240.77352	180.83196	A	1141.57095	571.28911	381.19517	286.14819	1142.55496	571.78112	381.52317	286.39420	15
10	790.35912	395.68320	264.12456	198.34524	791.34314	396.17521	264.45256	198.59124	A	1070.53383	535.77055	357.51613	268.38891	1071.51784	536.26256	357.84413	268.63492	14
11	847.38059	424.19393	283.13172	212.60061	848.36461	424.68594	283.45972	212.84661	G	999.49671	500.25199	333.83709	250.62963	1000.48072	500.74400	334.16509	250.87564	13
12	918.41771	459.71249	306.81076	230.35989	919.40173	460.20450	307.13876	230.60589	A	942.47524	471.74126	314.82993	236.37427	943.45925	472.23326	315.15794	236.62027	12
13	989.45483	495.23105	330.48980	248.11917	990.43885	495.72306	330.81780	248.36517	A	871.43812	436.22270	291.15089	218.61499	872.42213	436.71470	291.47890	218.86099	11
14	1060.49195	530.74961	354.16884	265.87845	1061.47597	531.24162	354.49684	266.12445	A	800.40100	400.70414	267.47185	200.85571	801.38501	401.19614	267.79986	201.10171	10
15	1117.51342	559.26035	373.17599	280.13381	1118.49744	559.75236	373.50400	280.37982	G	729.36388	365.18558	243.79281	183.09643	730.34789	365.67758	244.12082	183.34243	9
16	1188.55054	594.77891	396.85503	297.89309	1189.53456	595.27092	397.18304	298.13910	A	672.34241	336.67484	224.78565	168.84106	673.32642	337.16685	225.11366	169.08706	8
17	1259.58766	630.29747	420.53407	315.65237	1260.57168	630.78948	420.86208	315.89838	A	601.30529	301.15628	201.10661	151.08178	602.28930	301.64829	201.43462	151.32778	7
18	1330.62478	665.81603	444.21311	333.41165	1331.60880	666.30804	444.54112	333.65766	A	530.26817	265.63772	177.42757	133.32250	531.25218	266.12973	177.75558	133.56850	6

19	1387.64625	694.32676	463.22027	347.66702	1388.63027	694.81877	463.54827	347.91302	G	459.23105	230.11916	153.74853	115.56322	460.21506	230.61117	154.07654	115.80922	5
20	1474.67828	737.84278	492.23095	369.42503	1475.66230	738.33479	492.55895	369.67103	S	402.20958	201.60843	134.74138	101.30785	403.19359	202.10043	135.06938	101.55386	4
21	1561.71031	781.35879	521.24162	391.18304	1562.69433	781.85080	521.56963	391.42904	S	315.17755	158.09241	105.73070	79.54984	316.16156	158.58442	106.05871	79.79585	3
22	1632.74743	816.87735	544.92066	408.94232	1633.73145	817.36936	545.24867	409.18832	A					229.12953	115.06840	77.04803	58.03784	2
23									R					158.09241	79.54984	53.36899	40.27856	1

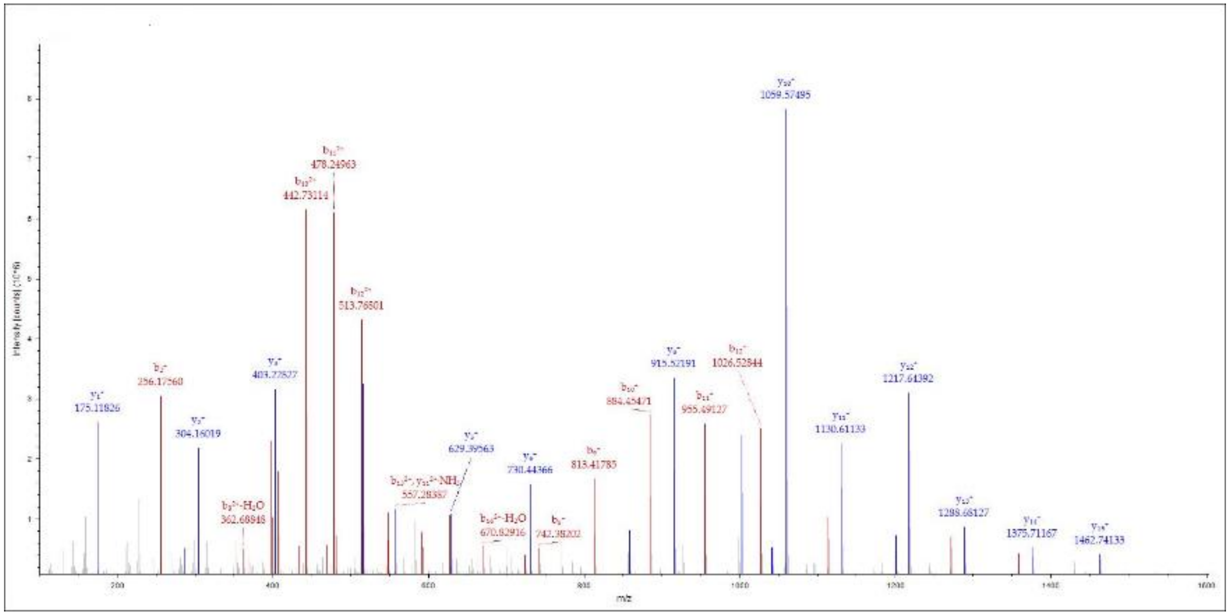

Figure S-5I The fragment peaks of peptide RVPGASSSAAAASSASAGSGQTIIVER.

#1	b-H_2O^+	b-H_2O^{2+}	b-H_2O^{3+}	b-NH_3^+	b-NH_3^{2+}	b-NH_3^{3+}	Seq.	y-H_2O^+	y-H_2O^{2+}	y-H_2O^{3+}	y-NH_3^+	y-NH_3^{2+}	y-NH_3^{3+}	#2
1				140.08185	70.54456	47.36547	R							27
2				239.15027	120.07877	80.38827	V	2314.16347	1157.58537	772.05934	2315.14748	1158.07738	772.38735	26
3				336.20304	168.60516	112.73920	P	2215.09505	1108.05116	739.03653	2216.07906	1108.54317	739.36454	25
4				393.22451	197.11589	131.74635	G	2118.04228	1059.52478	706.68561	2119.02629	1060.01678	707.01362	24
5				464.26163	232.63445	155.42539	A	2061.02081	1031.01404	687.67845	2062.00482	1031.50605	688.00646	23

6	550.30964	275.65846	184.10807	551.29366	276.15047	184.43607	S	1989.98369	995.49548	663.99941	1990.96770	995.98749	664.32742	22
7	637.34167	319.17447	213.11874	638.32569	319.66648	213.44675	S	1902.95166	951.97947	634.98874	1903.93567	952.47147	635.31674	21
8	724.37370	362.69049	242.12942	725.35772	363.18250	242.45742	S	1815.91963	908.46345	605.97806	1816.90364	908.95546	606.30607	20
9	795.41082	398.20905	265.80846	796.39484	398.70106	266.13646	A	1728.88760	864.94744	576.96738	1729.87161	865.43944	577.29539	19
10	866.44794	433.72761	289.48750	867.43196	434.21962	289.81550	A	1657.85048	829.42888	553.28834	1658.83449	829.92088	553.61635	18
11	937.48506	469.24617	313.16654	938.46908	469.73818	313.49454	A	1586.81336	793.91032	529.60930	1587.79737	794.40232	529.93731	17
12	1008.52218	504.76473	336.84558	1009.50620	505.25674	337.17358	A	1515.77624	758.39176	505.93026	1516.76025	758.88376	506.25827	16
13	1095.55421	548.28074	365.85626	1096.53823	548.77275	366.18426	S	1444.73912	722.87320	482.25122	1445.72313	723.36520	482.57923	15
14	1182.58624	591.79676	394.86693	1183.57026	592.28877	395.19494	S	1357.70709	679.35718	453.24055	1358.69110	679.84919	453.56855	14
15	1253.62336	627.31532	418.54597	1254.60738	627.80733	418.87398	A	1270.67506	635.84117	424.22987	1271.65907	636.33317	424.55788	13
16	1340.65539	670.83133	447.55665	1341.63941	671.32334	447.88465	S	1199.63794	600.32261	400.55083	1200.62195	600.81461	400.87884	12
17	1411.69251	706.34989	471.23569	1412.67653	706.84190	471.56369	A	1112.60591	556.80659	371.54015	1113.58992	557.29860	371.86816	11
18	1468.71398	734.86063	490.24285	1469.69800	735.35264	490.57085	G	1041.56879	521.28803	347.86111	1042.55280	521.78004	348.18912	10
1	1555.74	778.376	519.253	1556.73	778.868	519.581	S	984.547	492.777	328.853	985.531	493.269	329.181	9

9	601	64	52	003	65	53		32	30	96	33	30	96	
20	1612.76748	806.88738	538.26068	1613.75150	807.37939	538.58868	G	897.51529	449.26128	299.84328	898.49930	449.75329	300.17129	8
21	1740.82606	870.91667	580.94687	1741.81008	871.40868	581.27488	Q	840.49382	420.75055	280.83612	841.47783	421.24255	281.16413	7
22	1841.87374	921.44051	614.62943	1842.85776	921.93252	614.95744	T	712.43524	356.72126	238.14993	713.41925	357.21326	238.47794	6
23	1954.95781	977.98254	652.32412	1955.94183	978.47455	652.65213	I	611.38756	306.19742	204.46737	612.37157	306.68942	204.79538	5
24	2068.04188	1034.52458	690.01881	2069.02590	1035.01659	690.34682	I	498.30349	249.65538	166.77268	499.28750	250.14739	167.10069	4
25	2167.11030	1084.05879	723.04162	2168.09432	1084.55080	723.36962	V	385.21942	193.11335	129.07799	386.20343	193.60535	129.40600	3
26	2296.15290	1148.58009	766.05582	2297.13692	1149.07210	766.38382	E	286.15100	143.57914	96.05518	287.13501	144.07114	96.38319	2
27							R				158.09241	79.54984	53.36899	1

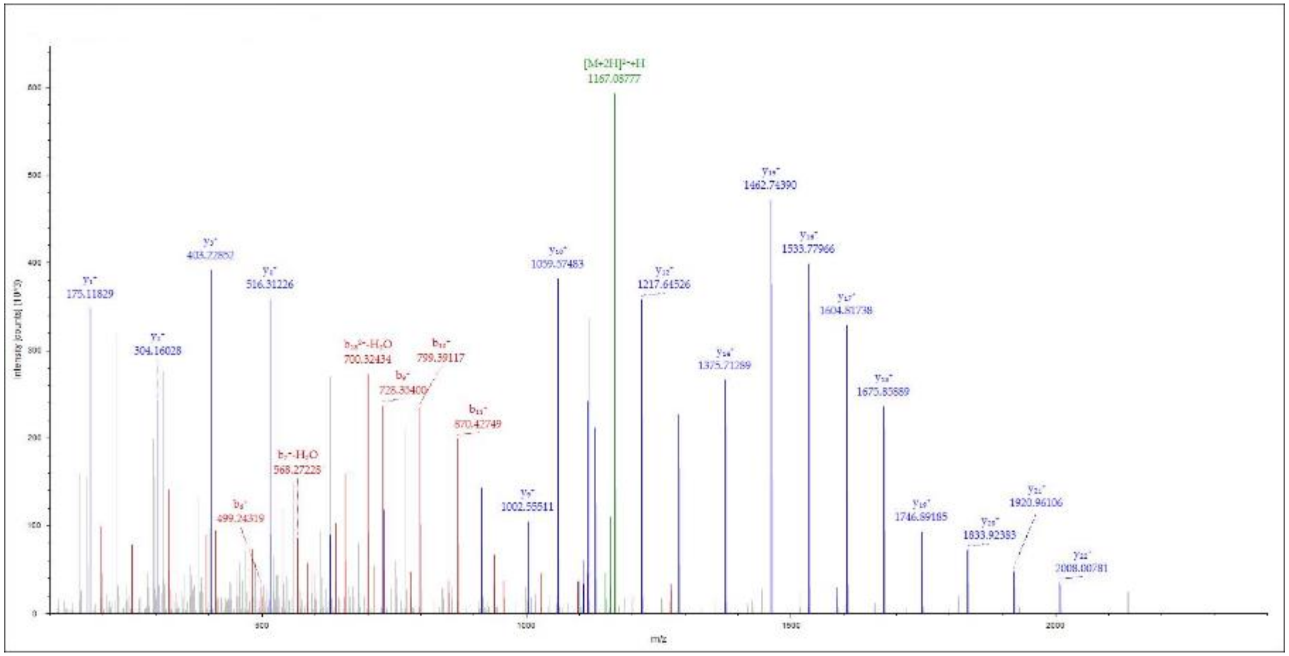

Figure S-5J The fragment peaks of peptide VPGASSSAAAASSASAGSGQTIIVER.

#1	b-H_2O^+	b-H_2O^{2+}	b-NH_3^+	b-NH_3^{2+}	Seq.	y-H_2O^+	y-H_2O^{2+}	y-NH_3^+	y-NH_3^{2+}	#2
1					V					26
2					P	2215.09505	1108.05116	2216.07906	1108.54317	25
3					G	2118.04228	1059.52478	2119.02629	1060.01678	24
4					A	2061.02081	1031.01404	2062.00482	1031.50605	23
5	394.20852	197.60790			S	1989.98369	995.49548	1990.96770	995.98749	22
6	481.24055	241.12391			S	1902.95166	951.97947	1903.93567	952.47147	21
7	568.27258	284.63993			S	1815.91963	908.46345	1816.90364	908.95546	20
8	639.30970	320.15849			A	1728.88760	864.94744	1729.87161	865.43944	19
9	710.34682	355.67705			A	1657.85048	829.42888	1658.83449	829.92088	18

10	781.38394	391.19561			A	1586.81336	793.91032	1587.79737	794.40232	17
11	852.42106	426.71417			A	1515.77624	758.39176	1516.76025	758.88376	16
12	939.45309	470.23018			S	1444.73912	722.87320	1445.72313	723.36520	15
13	1026.48512	513.74620			S	1357.70709	679.35718	1358.69110	679.84919	14
14	1097.52224	549.26476			A	1270.67506	635.84117	1271.65907	636.33317	13
15	1184.55427	592.78077			S	1199.63794	600.32261	1200.62195	600.81461	12
16	1255.59139	628.29933			A	1112.60591	556.80659	1113.58992	557.29860	11
17	1312.61286	656.81007			G	1041.56879	521.28803	1042.55280	521.78004	10
18	1399.64489	700.32608			S	984.54732	492.77730	985.53133	493.26930	9
19	1456.66636	728.83682			G	897.51529	449.26128	898.49930	449.75329	8
20	1584.72494	792.86611	1585.70896	793.35812	Q	840.49382	420.75055	841.47783	421.24255	7
21	1685.77262	843.38995	1686.75664	843.88196	T	712.43524	356.72126	713.41925	357.21326	6
22	1798.85669	899.93198	1799.84071	900.42399	I	611.38756	306.19742	612.37157	306.68942	5
23	1911.94076	956.47402	1912.92478	956.96603	I	498.30349	249.65538	499.28750	250.14739	4
24	2011.00918	1006.00823	2011.99320	1006.50024	V	385.21942	193.11335	386.20343	193.60535	3
25	2140.05178	1070.52953	2141.03580	1071.02154	E	286.15100	143.57914	287.13501	144.07114	2
26					R			158.09241	79.54984	1

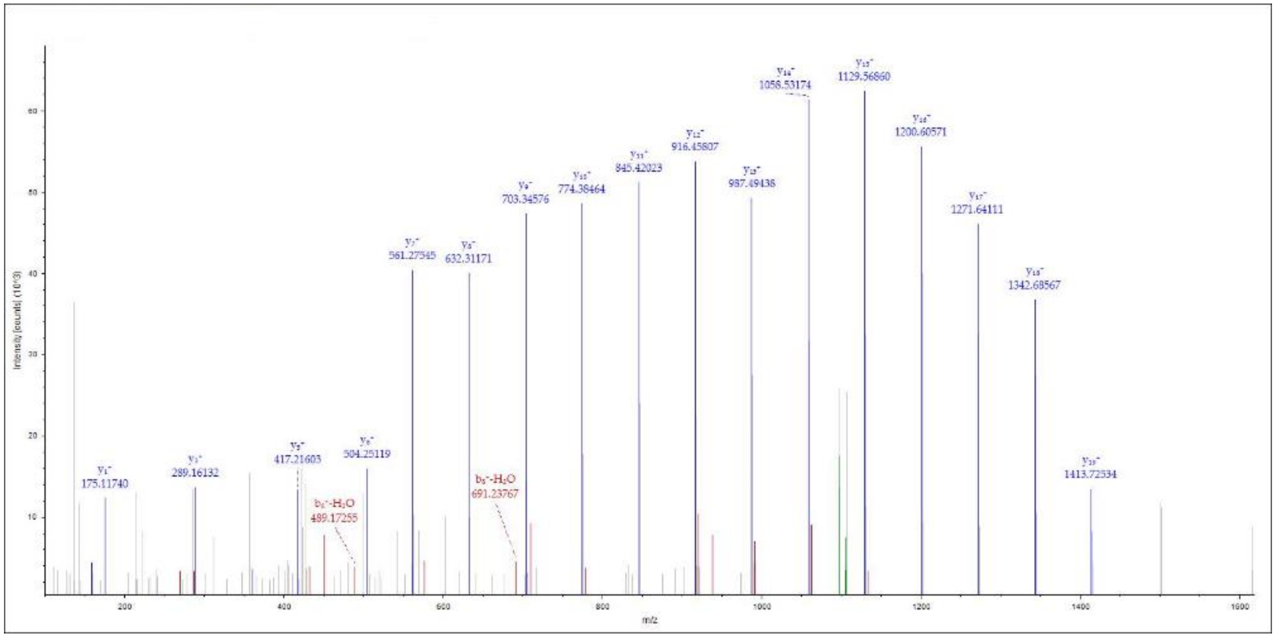

Figure S-6A The fragment peaks of peptide GDGGYGSDSAAAAAAAAAAAAGSGAGGR.

#1	b-H_2O^+	b-H_2O^{2+}	Seq.	y-H_2O^+	y-H_2O^{2+}	y-NH_3^+	y-NH_3^{2+}	#2
1			G					28
2	155.04513	78.02620	D	2133.95456	1067.48092	2134.93857	1067.97292	27
3	212.06660	106.53694	G	2018.92761	1009.96744	2019.91162	1010.45945	26
4	269.08807	135.04767	G	1961.90614	981.45671	1962.89015	981.94871	25
5	432.15139	216.57933	Y	1904.88467	952.94597	1905.86868	953.43798	24
6	489.17286	245.09007	G	1741.82135	871.41431	1742.80536	871.90632	23
7	576.20489	288.60608	S	1684.79988	842.90358	1685.78389	843.39558	22
8	691.23184	346.11956	D	1597.76785	799.38756	1598.75186	799.87957	21
9	778.26387	389.63557	S	1482.74090	741.87409	1483.72491	742.36609	20

10	849.30099	425.15413	A	1395.70887	698.35807	1396.69288	698.85008	19
11	920.33811	460.67269	A	1324.67175	662.83951	1325.65576	663.33152	18
12	991.37523	496.19125	A	1253.63463	627.32095	1254.61864	627.81296	17
13	1062.41235	531.70981	A	1182.59751	591.80239	1183.58152	592.29440	16
14	1133.44947	567.22837	A	1111.56039	556.28383	1112.54440	556.77584	15
15	1204.48659	602.74693	A	1040.52327	520.76527	1041.50728	521.25728	14
16	1275.52371	638.26549	A	969.48615	485.24671	970.47016	485.73872	13
17	1346.56083	673.78405	A	898.44903	449.72815	899.43304	450.22016	12
18	1417.59795	709.30261	A	827.41191	414.20959	828.39592	414.70160	11
19	1488.63507	744.82117	A	756.37479	378.69103	757.35880	379.18304	10
20	1559.67219	780.33973	A	685.33767	343.17247	686.32168	343.66448	9
21	1630.70931	815.85829	A	614.30055	307.65391	615.28456	308.14592	8
22	1687.73078	844.36903	G	543.26343	272.13535	544.24744	272.62736	7
23	1774.76281	887.88504	S	486.24196	243.62462	487.22597	244.11662	6
24	1831.78428	916.39578	G			400.19394	200.60061	5
25	1902.82140	951.91434	A			343.17247	172.08987	4
26	1959.84287	980.42507	G			272.13535	136.57131	3
27	2016.86434	1008.93581	G			215.11388	108.06058	2
28			R			158.09241	79.54984	1

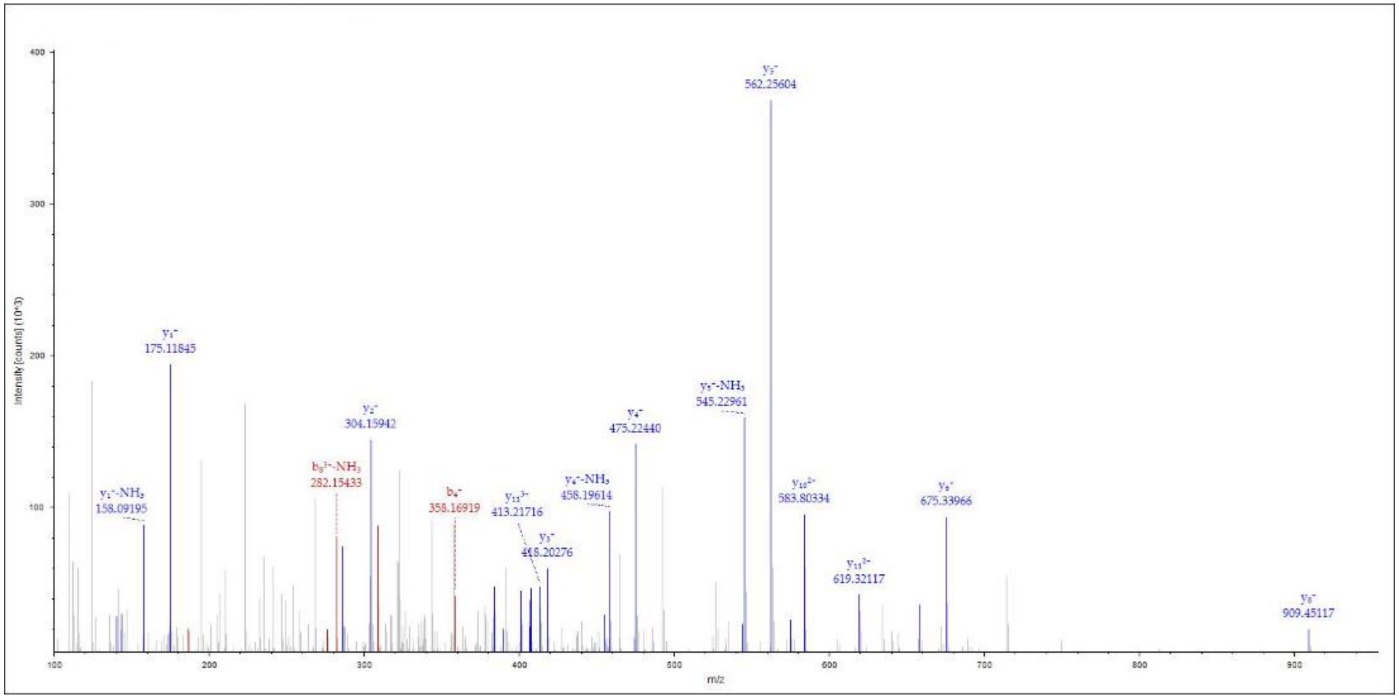

Figure S-6B The fragment peaks of peptide NAATRPHLSGNER.

#1	b-H_2O^+	b-H_2O^{2+}	b-H_2O^{3+}	b-H_2O^{4+}	b-NH_3^+	b-NH_3^{2+}	b-NH_3^{3+}	b-NH_3^{4+}	Seq.	y-H_2O^+	y-H_2O^{2+}	y-H_2O^{3+}	y-H_2O^{4+}	y-NH_3^+	y-NH_3^{2+}	y-NH_3^{3+}	y-NH_3^{4+}	#2
1					98.02366	49.51547	33.34607	25.26137	N									13
2					169.06078	85.03403	57.02511	43.02065	A	1290.66622	645.83675	430.89359	323.42201	1291.65023	646.32875	431.22160	323.66802	12
3					240.09790	120.55259	80.70415	60.77993	A	1219.62910	610.31819	407.21455	305.66273	1220.61311	610.81019	407.54256	305.90874	11
4	340.1	170.5	114.0	85.79	341.1	171.0	114.3	86.04	T	1148.	574.7	383.5	287.9	1149.	575.2	383.8	288.1	1

	6156	8442	5871	585	4558	7643	8671	185		59198	9963	3551	0345	57599	9163	6352	4946	0
5	496.26268	248.63498	166.09241	124.82113	497.24670	249.12699	166.42042	125.06713	R	1047.54430	524.27579	349.85295	262.64153	1048.52831	524.76779	350.18096	262.88754	9
6	593.31545	297.16136	198.44334	149.08432	594.29947	297.65337	198.77134	149.33032	P	891.44318	446.22523	297.81924	223.61625	892.42719	446.71723	298.14725	223.86226	8
7	730.37436	365.69082	244.12964	183.34905	731.35838	366.18283	244.45764	183.59505	H	794.39041	397.69884	265.46832	199.35306	795.37442	398.19085	265.79633	199.59906	7
8	843.45843	422.23285	281.82433	211.62007	844.44245	422.72486	282.15233	211.86607	L	657.33150	329.16939	219.78202	165.08833	658.31551	329.66139	220.11002	165.33434	6
9	930.49046	465.74887	310.83501	233.37807	931.47448	466.24088	311.16301	233.62408	S	544.24743	272.62735	182.08733	136.81731	545.23144	273.11936	182.41533	137.06332	5
10	987.51193	494.25960	329.84216	247.63344	988.49595	494.75161	330.17017	247.87944	G	457.21540	229.11134	153.07665	115.05931	458.19941	229.60334	153.40466	115.30531	4
11	1101.55486	551.28107	367.85647	276.14417	1102.53888	551.77308	368.18448	276.39018	N	400.19393	200.60060	134.06949	100.80394	401.17794	201.09261	134.39750	101.04994	3
12	1230.59746	615.80237	410.87067	308.40482	1231.58148	616.29438	411.19868	308.65083	E	286.15100	143.57914	96.05518	72.29321	287.13501	144.07114	96.38319	72.53921	2
13									R					158.09241	79.54984	53.36899	40.27856	1
